# Lactylation in health and disease: physiological or pathological?

**DOI:** 10.7150/thno.105353

**Published:** 2025-01-02

**Authors:** Lijun Zhao, Haonan Qi, Huiying Lv, Wenyue Liu, Rui Zhang, Angang Yang

**Affiliations:** 1 Key Laboratory of Holistic Integrative Management of Gastrointestinal Cancers and Department of Immunology, Fourth Military Medical University, Xi'an, Shanxi 710032, China.; 2 Henan Key Laboratory of Immunology and Targeted Drugs, Xinxiang Key Laboratory of Tumor Microenvironment and Immunotherapy, School of Medical Technology, Xinxiang Medical University, Xinxiang, Henan, China.

**Keywords:** Lactylation, Regulatory mechanism, Physiology, Pathology

## Abstract

Lactate is an indispensable substance in various cellular physiological functions and plays regulatory roles in different aspects of energy metabolism and signal transduction. Lactylation (Kla), a key pathway through which lactate exerts its functions, has been identified as a novel posttranslational modification (PTM). Research indicates that Kla is an essential balancing mechanism in a variety of organisms and is involved in many key cellular biological processes through different pathways. Kla is closely related to disease development and represents a potential and important new drug target. In line with existing reports, we searched for newly discovered Kla sites on histone and nonhistone proteins; reviewed the regulatory mechanisms of Kla (particularly focusing on the enzymes directly involved in the reversible regulation of Kla, including “writers” (modifying enzymes), “readers” (modification-binding enzymes), and “erasers” (demodifying enzymes); and summarized the crosstalk between different PTMs to help researchers better understand the widespread distribution of Kla and its diverse functions. Furthermore, considering the "double-edged sword" role of Kla in both physiological and pathological contexts, this review highlights the "beneficial" biological functions of Kla in physiological states (energy metabolism, inflammatory responses, cell fate determination, development, etc.) and its "detrimental" pathogenic or inducive effects on pathological processes, particularly malignant tumors and complex nontumor diseases. We also clarify the molecular mechanisms of Kla in health and disease, and discuss its feasibility as a therapeutic target. Finally, we describe the detection technologies for Kla and their potential applications in diagnosis and clinical settings, aiming to provide new insights for the treatment of various diseases and to accelerate translation from laboratory research to clinical practice.

## Introduction

Lactate was traditionally considered a metabolic waste product of glycolysis under low-oxygen conditions. However, the lactate shuttle hypothesis has reshaped this perspective, emphasizing lactate's critical roles in cellular functions, energy metabolism, and signal transduction 1. In recent years, increasing research has shown that lactate not only serves as an energy source for mitochondrial respiration but also plays vital roles in processes such as inflammation, wound healing, memory formation, neuroprotection, and pathological conditions like tumor growth and metastasis, influencing disease progression and prognosis 1, 2.

Lactylation (Kla) represents a key pathway for lactate's functions and was first identified on proteins several years ago. However, the biological significance of this modification remained unclear until 2019, when Zhao Yingming's team identified a mass shift of 72.021 Da on histone lysine residues. This matched the addition of a lactyl group to the ε-amino group of lysine residues, leading them to propose Kla as a novel type of protein post-translational modification (PTM) 3. Recent research suggests that Kla is a vital regulatory mechanism in various organisms, playing critical roles in processes such as cellular energy metabolism, inflammatory responses, cell fate determination, and development. Moreover, Kla interacts with tumor-related genes, influencing the expression and function of oncogenes. It regulates the tumor immune microenvironment, tumor cell metabolism, drug resistance, and autophagy, thereby impacting the progression of tumor and non-tumor diseases 1, 2, 4.

Despite these advances, the precise regulatory mechanisms and biological functions of Kla remain poorly understood, and its role in physiological and pathological processes is still under investigation. This review summarizes recent preclinical *in vivo* and *in vitro* studies on lactylation, systematically examining its regulatory roles and molecular mechanisms in health and disease. Additionally, we highlight key findings, unresolved issues, and propose potential strategies to guide future clinical research and treatment development.

## Lactylation modification sites

### Histone lysine lactylation sites

Lactylation was first identified on lysine residues of human histones, with Professor Zhao discovering 28 Kla sites on core histones in 2019, mainly H3 and H4 3. Since then, an increasing number of histone Kla modification sites have been identified (Table 1). Research indicates that histone Kla mainly occurs on lysine in H2A, H2B, H3, and H4, particularly H3K18 5. H3K18 lactylation is typically enriched in gene promoter and enhancer regions, serving not only as an indicator of active gene expression but also being closely linked to various physiological and pathological processes, including gene transcription regulation, stem cell and embryonic development, macrophage polarization, and tumorigenesis 5. In addition to H3K18, other histone lactylation sites with regulatory functions have been reported, including H2BK6, H3K9, H3K14, H3K23, H3K56, H4K5, H4K80, and H4K12. These histone Kla modifications have been shown to be involved in the regulation of various cancer types, including lung cancer, prostate cancer, kidney cancer, colon cancer, liver cancer, and melanoma 6. However, most studies to date have focused on specific gene sets, and it remains unclear whether histone Kla generally suppresses or enhances gene expression. In summary, histone Kla modifications are widely present and conserved, closely associated with diseases, and holds potential as a therapeutic target.

### Nonhistone lysine lactylation sites

In recent years, research has shown that Kla modifications are not limited to histones but are also widely present on nonhistone lysine residues (Table 2). Nonhistone Kla may have various effects, influencing protein stability, localization, structure, interactions, or functions by enhancing or inhibiting the original functions of these nonhistone proteins, and it is associated with multiple physiological and pathological contexts 4. For instance, Miao *et al.* reported that hypoxia-induced glycolysis promotes the Kla of β-catenin, further increasing its protein stability and expression, which exacerbates the malignant behavior of colorectal cancer (CRC) cells 7. Sun *et al.* discovered that copper ions regulate METTL16 K229 Kla by modulating lactylation and delactylation enzymes, such as SIRT2, thereby affecting its activity. Lactylated METTL16 leads to methylation at the FDX1 602 site, promoting FDX1 expression and resulting in copper-induced cell death 8. Gu *et al.* demonstrated that lactate regulates the generation of Treg cells through the lactylation of Lys72 in MOESIN, thereby improving the interaction between MOESIN and transforming growth factor β (TGF-β) receptor I and downstream SMAD3 signaling 9. Additionally, studies have shown that enzymes involved in metabolic processes, particularly glycolysis, are often subject to Kla modifications. For example, the key glycolytic enzyme pyruvate kinase M2 (PKM2) can undergo lactylation at the K62 site 10. Fructose-bisphosphate aldolase A (ALDOA) is lactylated at the K147 residue. The glycolytic enzyme enolase is lactylated at the K343 site, affecting substrate-enzyme interactions 11. Dehydrogenase reductase 7 can also be lactylated at K321, and this phenomenon is commonly observed in various human tissues, including the retina, spinal cord, liver, testis, ovary, and prostate 11. In summary, the discovery of these modification sites indicates that Kla has a broader regulatory role in biological systems than previously thought, highlighting its significant research value in biological process regulation.

## Regulatory mechanisms of lactylation

Lactate typically occurs in three stereoisomeric forms: D-lactic acid, L-lactic acid, and racemic DL-lactic acid (due to asymmetric carbon atoms). Lactylation encompasses three structurally similar but stereochemically distinct isomers: L-lactylation (K_L-La_), D-lactylation (K_D-La_) and N-ε-(carboxyethyl)-lysine (K_ce_) 12.

Under conditions of high glycolysis (e.g., hypoxia, cancer metabolism), elevated lactate production favors the formation of K_L-La_ or K_D-la_. Among these, L-lactic acid is the predominant isomer *in vivo*, while K_L-La_ serves as an important modification responsive to glycolysis 12. K_L-La_ involves attaching an L-lactyl group (which has polarity and hydroxyl groups) to lysine residues, enhancing their polarity and solubility. This modification can influence protein folding, receptor binding, structure, stability, and interactions with other molecules. Similarly, K_D-La_ entails attaching a D-lactyl group, which differs structurally from L-lactyl, to lysine residues, affecting protein stability, interactions, and biological activity. However, due to the stereochemical properties of the D-lactyl group, K_D-La_ may exhibit unique spatial selectivity. Currently, K_D-La_ (produced through a nonenzymatic process involving lactylated glutathione) has been observed only when the glycolytic enzyme system is impaired, and it is associated with microbial metabolism or metabolic disorders 13. In metabolically balanced states, Kce may dominate. Unlike K_L-La_, Kce involves adding a carboxyethyl group (acidic) to lysine's amino group, affecting enzyme activity, binding capacity, or intracellular localization of proteins. This modification alters lysine's charge state and polarity, impacting protein structure, active sites, and interactions, and is associated with oxidative stress, inflammation, and metabolic regulation 12. In summary, these three lysine modifications alter the chemical properties and spatial configurations of lysine, potentially leading to different effects on protein function. The specific impact depends on the protein type, modification site, and cellular environment.

**Figure 1:**
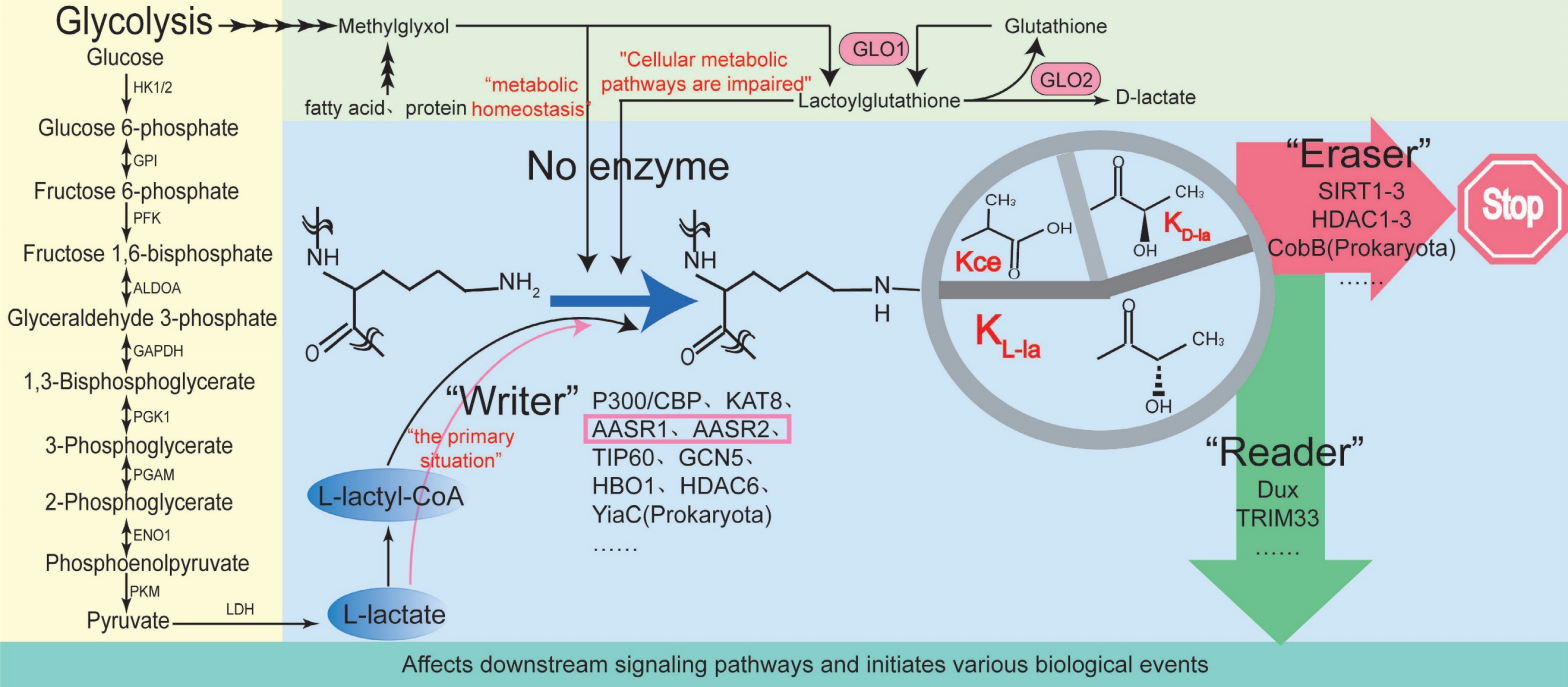
Regulatory mechanisms of of lactylation. Lysine l-lactylation (K_L-la_) is a novel protein posttranslational modification (PTM) driven by l-lactate. In addition to K_L-la_, it has two structurally similar but stereochemically distinct isomers: *N*-ε-(carboxyethyl)-lysine (K_ce_) and D-lactyl-lysine (K_D-la_). Depending on the different precursors, the Kla process can be enzymatic or nonenzymatic. Enzyme-dependent Kla, particularly Kl-la, has been widely studied. In enzyme-dependent Kla, the “writer” (modifying enzymes) uses endogenous or exogenous l-lactic acid as a substrate to transfer lactyl groups from lactyl-CoA to lysine residues on histones or non-histones, altering protein structure and function. The “reader” (modification-binding enzymes) recognizes Kla changes, influencing downstream signaling pathways and triggering biological events. When signal transduction ends, “erasers” (demodifying enzymes) remove lactyl groups from target proteins, halting the Kla cycle and mitigating the lasting effects of lysine Kla.

The Kla process can be enzymatic or nonenzymatic, depending on the precursors (Fig. 1) 14. Enzymatic Kla, particularly K_L-La_, is widely studied. In enzymatic Kla, the “writer” (modifying enzyme) transfers lactyl groups from lactyl-CoA to lysine residues on histones or non-histones, using endogenous or exogenous l-lactic acid, which alters protein structure and function. The “reader” (modification-binding enzymes, such as Dux 15 and TRIM33 16) recognizes Kla changes, influencing signaling pathways and triggering biological events. When signal transduction ends, “erasers” (demodifying enzymes) remove lactyl groups, halting the Kla cycle and reducing its lasting effects.

According to reports, enzyme-dependent lactylation is dynamically regulated by classical histone acetyltransferases 17, 18. Acetyltransferase-mediated Kla modification typically depends on the enzyme's activity and substrate specificity. Under conditions of abundant nutrients and balanced energy metabolism (such as during cell proliferation, differentiation, or in the tumor microenvironment), acetyltransferase-mediated lactylation predominates. Acetyltransferases involved in Kla modifications can precisely target lactylation modifications to specific proteins 3. For instance, p300, a classical histone acetyltransferase, serves as a “writer” for YY1 K-la, adding lactyl groups from lactyl-CoA to lysine residues, influencing cellular inflammation 17. Additionally, p300 serves as a “writer” for Kla on the YTHDF2 promoter, promoting the degradation of PER1 and TP53 mRNA, accelerating ocular melanoma onset 18. P300 also functions as a “writer” for the Kla of APCO2-K70, enhancing its Kla and promoting lipolysis 19. TIP60, another acetyltransferase, catalyzes the Kla of NBS1 at K388, directly modifying the NBS1 protein, with enhanced effects under lactate and cisplatin treatment 20. High lactate levels in tumors act as signaling molecules, mediating PIK3C3/VPS34 Kla via TIP60 at lysines 356 and 781, which enhances autophagy and promotes tumor progression 21. Xie *et al.* discovered that KAT8, a lysine acetyltransferase, can act as a “writer” for panlactylation, installing Kla on numerous protein substrates involved in various biological processes 22. Furthermore, recent studies identified ACSS2 and KAT2A as previously uncharacterized lactate coenzyme A synthetase and transferase, respectively. KAT2A facilitates lactate transfer to histone H3, activating Wnt/β-catenin, NF-κB, and PD-L1, promoting brain tumor growth and immune evasion. The interaction between ACSS2 and KAT2A, combined with peptide blockade and anti-PD-1 antibody treatment, produces an additive tumor-suppressive effect 23. HDAC6, a deacetylase, also acts as a lactyltransferase, catalyzing the Kla of α-tubulin lysine 40 in soluble microtubule dimers. By competing with acetylation (Kac) at the same residue, it links cellular metabolism and cytoskeletal function through the regulation of microtubule dynamics. Interestingly, HDAC6 mediated lactylation is reversible, depending on lactate concentration. HDAC6 primarily lactylates α-tubulin under high lactate levels 24. Moreover, deacetylases like HDACs and sirtuins can remove protein Kla 25, 26. According to reports, HDAC3 is an effective “eraser” for K-la, interacting directly with NBS1 [20]and APCO2-K70 19 to remove Kla and inhibit its expression. SIRT1 can eliminate Kla of CNPY3, promoting lysosomal rupture and triggering specific pyroptosis in prostate cancer cells 26. SIRT3 can remove Kla from CCNE2, thereby regulating the cell cycle and hindering the progression of HCC 27. p300 and SIRT1 function as lactyltransferase and delactylase for α-MHC, respectively. Reducing lactate production through chemical or genetic manipulation can decrease α-MHC Kla, impairing the interaction between α-MHC and titin, which exacerbates heart failure 28.

Notably, no acetyltransferase has been identified with strict specificity for Kla, making it difficult to define the precise conditions for efficient catalysis 29. Specifically, the ability of acetyltransferases to distinguish between acetyl-CoA and lactyl-CoA primarily depends on enzyme structure, active site specificity, chemical environment affinity, and substrate kinetic differences. In the case of acetyl-CoA, the simple structure of the acetyl group (CH3CO-) facilitates the formation of specific hydrogen bonds and hydrophobic interactions, making it more adaptable to the enzyme's binding pocket. With respect to lactyl-CoA, the lactyl group (CH3CHOHCO-) is larger and more polar compared to the acetyl group, which may hinder its binding to certain acetyltransferases due to steric hindrance or insufficient affinity. The additional hydroxyl group may also interfere with this binding or require specific amino acid residues to stabilize its structure. Nevertheless, some studies suggest that certain acetyltransferases specialize in Kla or Kac through differences in active site residues, achieving functional differentiation. Furthermore, the catalytic selectivity of acetyltransferases for the two CoA derivatives is likely determined by intracellular concentrations and regulatory mechanisms. Acetyl-CoA generally exists at much higher intracellular concentrations, which may make it the preferred substrate. In contrast, endogenous lactyl-CoA levels in cancer cells are extremely low, around 0.011 pmol per 10⁶ cells, about 1,000 times lower than acetyl-CoA levels in mammalian cells 30. This low concentration, combined with competitive inhibition, may hinder the ability of proposed acetyltransferases such as p300, CREB-binding protein (CBP) and TIP60 to effectively transfer lactyl groups to target proteins *in vivo* 29. The low levels of lactylation also complicate distinguishing whether lactylation serves a primary regulatory function or is merely a secondary outcome of other metabolic processes 29. Additionally, the concentrations of lactyl-CoA used in *in vitro* experiments are not well-documented in the literature, and key enzymatic parameters such as the dissociation constant (Kd) and Michaelis constant (Km) remain unavailable, raising concerns about whether these experiments accurately reflect *in vivo* conditions 30. In summary, the crosstalk between Kla and Kac plays a key role in metabolism and epigenetics, warranting further investigation.

Besides acetyltransferases, mitochondrial alanyl-tRNA synthetase 1 (AARS1) and 2 (AARS2) also act as intracellular lactate sensors and lactate transferases 31. AARS is a key enzyme class responsible for attaching amino acids to their tRNAs. Under stress (e.g., oxidative stress or changes in protein synthesis), AARS activity quickly adjusts to amino acid and lactate fluctuations, potentially leading to lactylation of proteins involved in translation regulation 31. Recent evidence shows that, unlike acetyltransferases, AARS1 and AARS2 act as lactate sensors and lactyltransferases, using L-lactate, not lactyl-CoA, as a substrate, leading to lactylation of a wide range of proteins, including p53, Yap, and mitochondrial proteins 32-34. This is the first report in over half a century of a non-CoA-dependent catalytic reaction since acetylation's discovery, where glucose-derived lactate + ATP is fully utilized and covalently added to proteins, altering the function of key proteins 31.

Furthermore, Wang *et al.* found that GCN5, a lactylation-modifying enzyme, regulates target gene transcription, providing new insights into the interaction between the metabolome, epigenome, and immune response after myocardial infarction 35. YiaC, a member of the GCN5-related N-acetyltransferase (GNAT) family, is a lysine lactylase that catalyzes Kla in cells 36. Niu *et al.* showed that HBO1 has Kla catalytic activity, regulating 95 Kla sites, especially H3K9la. Additionally, the scaffold proteins JADE1 and BRPF2 enhance HBO1's catalytic activity toward H3K9la 37.

Nonenzymatic lactylation is the reaction between lactate and substrates without enzyme catalysis. It depends on environmental factors like pH, temperature, and substrate concentration 13. Under conditions of high glycolytic activity or vigorous lactate production, nonenzymatic Kla may become predominant. However, there is currently limited research on this topic, primarily focusing on lysine K_D-la_ and the formation of Kce through reactions with lysine. K_D-la_ forms through a nonenzymatic reaction between S-D-lactyl glutathione (LGSH), produced by the glyoxalase pathway, and proteins. Gaffney *et al.* showed that lactylation in cells depends on LGSH and GLO2 regulation at the K94 site of PGK1 13. Additionally, in the glyoxalase pathway, the high reactivity of the glycolytic byproduct methylglyoxal (MGO) allows it to react with various protein residues, including cysteine, arginine, and lysine. Among these, the Kce formed through reactions with lysine has been identified in cells, although its levels are lower than those of MGO-derived arginine residue modifications 13. In summary, lactylation regulation varies under different cellular conditions. Exploring lactylation regulation holds promise as a therapeutic target.

## Crosstalk between lactylation and other protein posttranslational modifications

**Figure 2:**
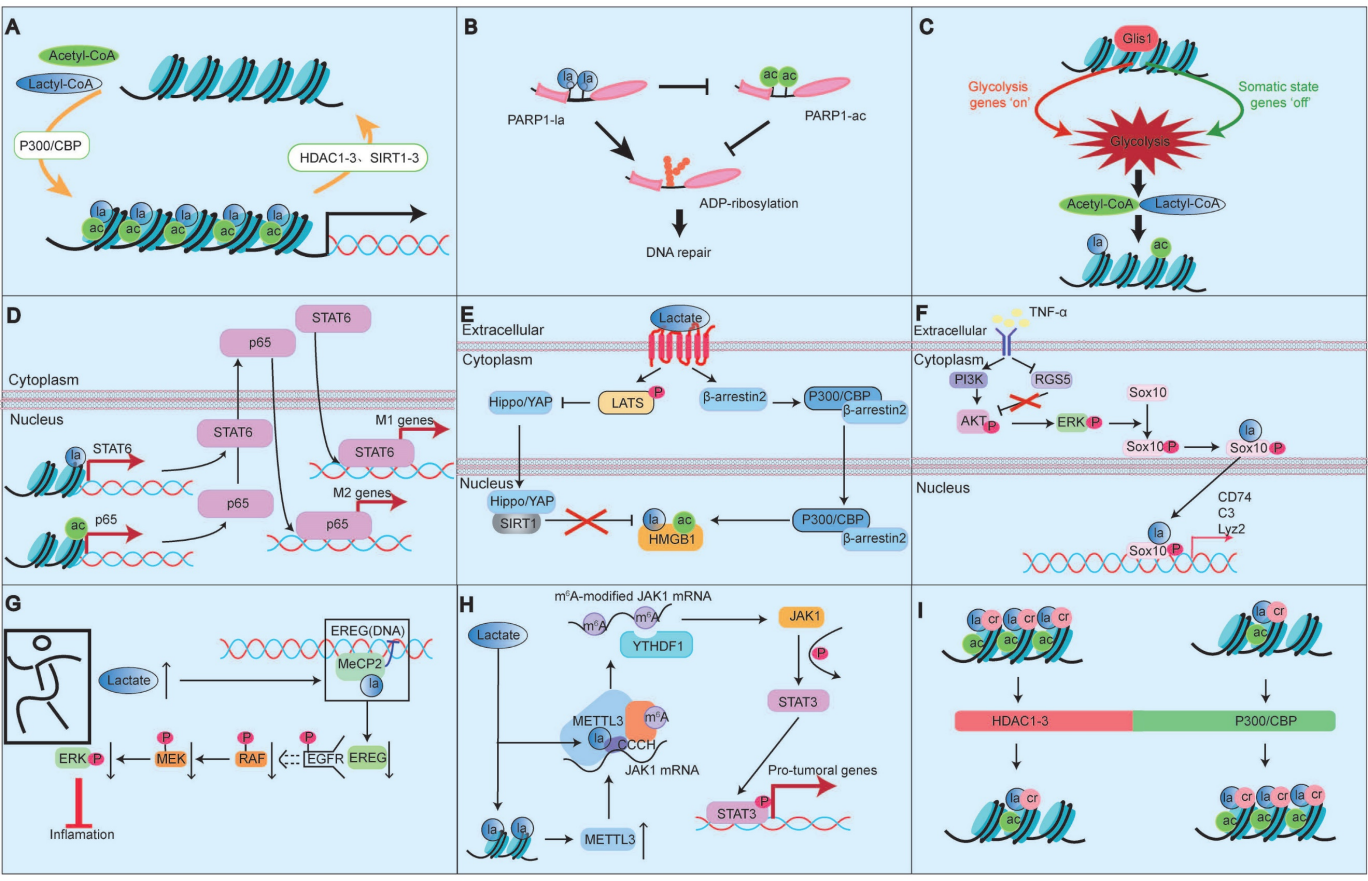
Crosstalk between lactylation and other post-translational modifications. The vast majority of PTMs do not exist independently. Instead, any two or more different PTMs can interact, where these combined PTM states can reinforce or inhibit each other. Notably, there is a high degree of similarity and coordination between lactylation and acetylation, and their crosstalk is an important process connecting metabolism and epigenetics (A-E). Besides acetylation, there is considerable research on the crosstalk between lactylation and phosphorylation (F-H). Other PTMs, such as crotonylation, butyrylation and succinylation, have also been reported to interact with lactylation. For instance, it has been observed that crotonylation and lactylation can occur on all core histones and share the most common modification sites with histone lysine acetylation (G).

PTMs are crucial epigenetic regulators in processes such as DNA replication, transcription, and cell differentiation, and play a key role in protein function 6. The most common PTMs include methylation, acetylation, phosphorylation, ubiquitination, SUMOylation, glycosylation, butyrylation, succinylation, and propionylation. Recent studies have shown that the vast majority of PTMs do not exist independently. Instead, any two or more different PTMs can interact, where these combined PTM states can reinforce or inhibit each other (Fig. 2) 6.

Among them, Kla and Kac exhibit significant similarities and coordination, with their crosstalk serving as a crucial link between metabolism and epigenetics. For instance, Li *et al.* reported that Gli-similar transcription factor 1 (Glis1) enhances levels of acetyl-CoA and lactate, driving histone Kac and Kla by activating glycolytic genes and increasing glycolytic flux 38. Yang *et al.* reported that in mixed bacterial sepsis, lactate simultaneously affects the Kla and Kac of macrophage HMGB1. Macrophages uptake extracellular lactate via monocarboxylate transporters, promoting HMGB1 Kla through a p300/CBP-dependent mechanism 39. Lactate also stimulates HMGB1 Kac via GPR81 through Hippo/YAP-mediated SIRT1 inhibition and β-arrestin2-mediated p300/CBP recruitment to the nucleus 39. Lu *et al.* reported that histone H3 lactylation synergistically promotes NF-κB and STAT6 transcription, influencing macrophage differentiation 40. Li *et al.* also noted that classic acyltransferases such as p300 can catalyze both Kac and Kla of transcription factors, histones, and other nuclear proteins in macrophages and iPSCs 41. However, some studies have pointed out the competitive relationship between Kla and Kac. For example, Sun *et al.* demonstrated that Kla and Kac can occur on the same residue within the self-modification domain of poly (ADP-ribose) polymerase 1(PARP1), where Kla may competitively inhibit Kac, restoring the ADP‒ribosyltransferase activity of PARP1 and promoting DNA repair while regulating pluripotency gene expression 42. Notably, histones Kla and Kac exhibit different temporal dynamics, with varying cellular responses to different stimuli. For example, under hypoxic conditions, Kla levels increase in both human HeLa cells and mouse macrophages, but Kac levels decrease in HeLa cells while remaining unchanged in mouse macrophages 3.

In addition to Kac, the crosstalk between Kla and phosphorylation has been studied extensively, often functioning synergistically. For example, Maschari *et al.* reported that sodium lactate treatment resulted in a dose-dependent relationship between protein Kla and IRS-1 serine 636 phosphorylation 43. Xu *et al.* demonstrated that tumor necrosis factor α (TNF-α) induces Sox10 Kla through a phosphorylation-dependent mechanism involving the PI3K/AKT pathway, affecting vascular smooth muscle cell transdifferentiation 44. Wang *et al.* reported that methyl-CpG binding protein 2 (Mecp2) k271la inhibits epithelial regulator protein expression by altering epidermal growth factor receptor phosphorylation levels, impacting the mitogen-activated protein kinase (MAPK) signaling pathway and improving atherosclerosis 45. Ma *et al.* found that methylthio-methane enhances H3K18la-specific target gene expression during *Staphylococcus aureus* infection, promoting STAT3 phosphorylation 46. Xiong *et al.* showed that lactate promotes METTL3 expression through H3K18la, increasing downstream signaling and increasing STAT3 phosphorylation levels 47.

Other PTMs, such as crotonylation, butyrylation and succinylation also interact with Kla. Studies indicate that crotonylation and Kla can occur on nearly all core histones and share modification sites with histone lysine Kac 6, 48. Kla may be related to butyrate-mediated butyrylation 48. In breast cancer treated with catalpol, Kac, 2-hydroxyisobutyrylation, and Kla were significantly increased, whereas succinylation, propionylation, and phosphorylation were significantly decreased, suggesting that catalpol may inhibit breast cancer progression by regulating different types of PTMs 49.

In summary, Kla is an important balancing mechanism in various organisms and has a complex relationship with other types of modifications. The crosstalk between Kla and other PTMs in different diseases, the potential synergistic or competitive relationships among various modifications in regulating protein function, and whether the ratio of different PTMs affects disease progression and prognosis, warrant further exploration.

## "Beneficial" regulation of lactylation in biological processes

**Figure 3:**
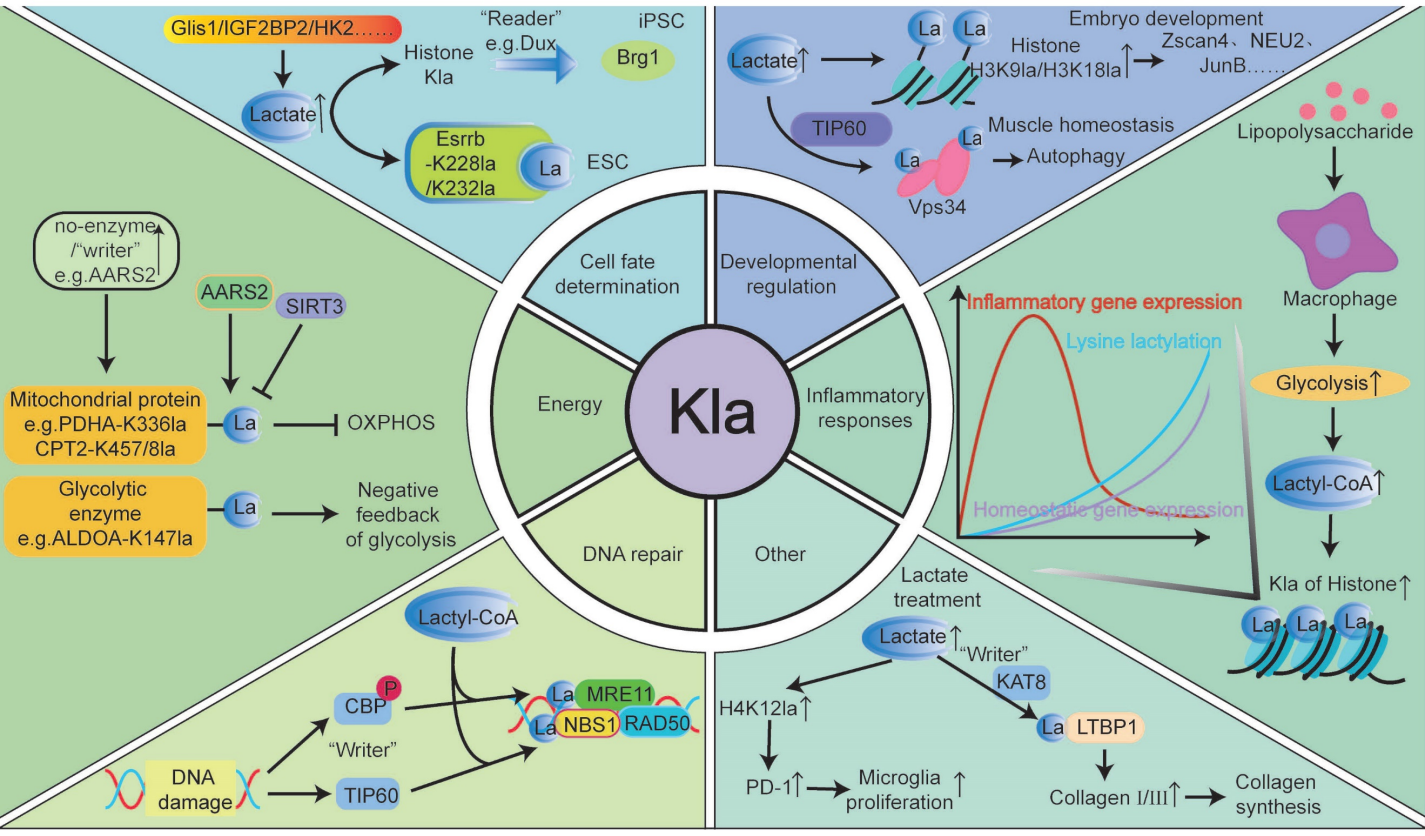
The “Beneficial” regulation of lactylation in biological processes. Lactylation serves as a “beneficial” modification, playing a significant role in various biological processes. For instance, in eukaryotes, lactate produced during glycolysis can regulate multiple metabolic pathways through Kla. In addition to mediating glycolysis, hypoxia can trigger mitochondrial protein lactylation, limiting oxidative phosphorylation. Kla modification also influences specific inflammatory signaling pathways and immune cell interactions, thereby modulating the intensity of inflammatory responses, immune cell cluster formation, and coordination of immune responses. Lactylation links metabolism, transcription, and epigenetics, regulating gene expression at the chromosomal level and impacting cell fate. It enhances pre-implantation embryo development, promotes transcriptional elongation, and plays a critical role in muscle generation and bone formation. Additionally, lactylation participates in DNA damage repair, holds potential for skin rejuvenation, and contributes to optimizing immune function. While its roles are promising, further research is needed to fully understand these processes.

Recent research highlights Kla, mediated by lactate, as a potentially beneficial substance involved in various biological processes, including energy metabolism, inflammatory responses, cell fate determination, and development (Fig. 3). Hereafter, we review the roles and mechanisms of Kla in human health and physiology.

### Energy metabolism

Metabolism is essential for life, as cells absorb nutrients to meet energy needs. Glucose is a key source of lactate in cells, and the lactate produced during glycolysis significantly influences various metabolic pathways, playing a vital role in cellular regulation 3. For example, Wan *et al.* found that Kla, resulting from lactate accumulation, inhibits glycolytic feedback by covalently modifying upstream enzymes. When the glycolytic pathway is overactivated and produces excessive lactate, Kla at K147 reduces ALDOA activity, decreasing glycolytic flux via a “negative feedback loop” 11. Gaffney *et al.* confirmed that Kla-modified proteins are enriched in the glycolytic pathway, and non-enzymatic Kla reactions using LGSH as a substrate also occurring on histones and non-histones 13. Jia *et al.* found that under nutrient deprivation, ULK1 can directly interact with the activated glycolytic enzyme LDHA, phosphorylate serine 196, and promote lactate production. Lactate mediates Vps34 lactylation (lysine 356 and lysine 781) via TIP60, thereby enhancing autophagy and glycolysis 21. In addition to mediating glycolysis, Zhao *et al.* proposed that hypoxia can induce mitochondrial protein lactylation to limit oxidative phosphorylation (OXPHOS). The study identified AARS2 as a lysine lactyltransferase, with its proteasomal degradation enhanced through proline 377 hydroxylation catalyzed by oxygen-sensing hydroxylase PHD2 33. Under hypoxia, AARS2 accumulation leads to lactylation of PDHA1 (lysine 336) and CPT2 (lysines 457/8), inactivating both enzymes and suppressing OXPHOS by limiting acetyl-CoA flux from pyruvate and fatty acid oxidation 33. And this lactylation can be reversed by SIRT3, reactivating OXPHOS 33.

### Inflammatory responses

Kla plays crucial roles in regulating immune cell activity, inflammatory responses, and interactions between immune cells 50, 51. Modifications in Kla can influence specific inflammatory signaling pathways and modulate immune cell interactions, which in turn regulates inflammation intensity, immune cell cluster formation, and immune response coordination 3. Macrophages, which are highly adaptable cells in the innate immune system, are essential in inflammatory responses. In the early stages of tissue damage, M1 macrophages initiate inflammation and eliminate external threats. Later, they polarize to the M2 phenotype to clear apoptotic cells and resolve inflammation 3. Zhang *et al.* found that in the later stages of M1 macrophage polarization, histone Kla significantly increases on the promoters of M2-like genes, suggesting that histone Kla may act as a lactate clock to promote the transition of macrophages from an inflammatory phenotype to a homeostatic phenotype. This transition occurs in the later stages of inflammation, which may be related to wound healing 3. Additionally, Zhang *et al.* observed that during bacterial infections, H3K18la enrichment at the promoters of M2-like genes, such as Arg1 and VEGFα, enhances gene expression, promoting M1-to-M2 macrophage conversion in the late polarization stage. This prevents further inflammation-induced damage 3. Irizarry-Caro *et al.* found that BCAP deficiency disrupts FOXO1 and GSK3β inactivation, resulting in heightened inflammation, impaired aerobic glycolysis, and reduced lactate production, resulting in decreased histone lactylation. Adding exogenous lactate to BCAP-deficient bone marrow-derived macrophages (BMDMs) restored histone lactylation and promoted the transition from inflammatory to reparative macrophage characteristics 50. Ma *et al.* reported that in macrophages from a peritonitis mouse model, MSM increased H3K18la levels, promoting the expression of M2 markers such as Arg1 51. This conferred protective effects against methicillin-resistant *Staphylococcus aureus* infections, suggesting a potential therapeutic strategy for addressing global drug-resistant infections 51.

### Cell fate determination

Kla links metabolism, transcription, and epigenetics, regulating gene expression at the chromosomal level and influencing cell fate. For instance, Dong *et al.* demonstrated that protein lactylation plays a key role in ESC self-renewal and extra-embryonic endoderm (XEN) differentiation. Their study showed that Esrrb, a nuclear receptor involved in pluripotency and XEN differentiation, is lactylated at K228 and K232. In the absence of LIF in ESCs or during XEN differentiation, Esrrb lactylation enhances its activity by increasing its binding to target genes, thereby promoting ESC self-renewal 52. Hu *et al.* revealed that during the early stages of induced pluripotent stem cell (iPSC) reprogramming, Dux acts as a histone lactylation reader. It activates a metabolic-lactylation-mesenchymal-epithelial transition network via Brg1, improving reprogramming efficiency through metabolic switches and recruiting p300 via its C-terminal domain 15. Li *et al.* found that histone lactylation significantly enhances stem cell survival, self-renewal, and reprogramming. In early reprogramming, Glis1 binds directly to and opens chromatin of glycolytic genes, increasing glycolysis. Elevated acetyl-CoA and lactate levels promote H3K27ac and H3K18la expression, facilitating senescent cell reprogramming and improving genomic stability 53. Additionally, Zhou *et al.* identified IGF2BP2 as an m6A-binding protein regulating glycolysis by modulating ALDOA expression, mediating histone Kla, and enhancing hepatic stellate cell (HSC) activation 54. Rho *et al.* found that lactate produced by activated HSCs induces hexokinase 2 (HK2) expression, determining HSC fate. HK2 deletion or inhibition of lactate production and histone Kla reduces HSC activation, while exogenous lactate (but not acetate) restores the activated phenotype and impacts HSC fate 55.

### Developmental regulation

Lactylation enhances preimplantation embryonic development, promotes transcriptional elongation, and plays a crucial role in this process. Yang *et al.* demonstrated that appropriate lactate concentrations at the fetal-maternal interface act as embryo-derived signals, promoting lactylation of endometrial histones. This modification regulates redox homeostasis, apoptosis, cell proliferation, cell adhesion, and immune tolerance in the endometrium, transforming it into a receptive state and offering insights into improving implantation outcomes 56. The study further highlighted that during pregnancy, increased histone H3K18la and lactate levels maintain glutathione-based redox homeostasis and apoptosis balance, both essential for successful embryo implantation 56. Yang *et al.* also found that under hypoxic conditions, histone Kla levels in oocytes and preimplantation embryos are significantly reduced, impairing developmental potential 57. Tian *et al.* confirmed that lactate upregulates cleavage-stage embryonic genes, such as the Zscan4 gene family, in embryonic stem cells. Lactate also promotes H3K18la in reproductive germline and cleavage-stage embryonic genes, enhancing transcriptional elongation 58. Zhao *et al.* noted that in the absence of lactate, H3K18la modifications are significantly reduced, suggesting that lactate primarily affects early embryonic development via H3K18la rather than H3K27ac modifications 59. Additionally, Yang *et al.* showed that inhibiting LDHA activity reduces lactate levels and histone Kla, thereby impairing preimplantation embryonic development 60. Merkuri *et al.* reported that glycolysis-regulated histone Kla integrates the metabolic state of embryonic cells with chromatin organization and gene regulatory network activation 61. Lactylation marks are enriched in glycolytic embryonic tissues, such as neural crest and precursor mesenchymal mesoderm 61. In summary, these findings demonstrate how histone Kla links cellular metabolism with developmental GRNs, providing precise insights for clinical interventions to improve pregnancy outcomes in natural conception and assisted reproductive technologies.

Lactate promotes osteoblast differentiation and bone formation. Maschari *et al.* first identified Kla in human skeletal muscle, linking its levels to osteoblast differentiation and bone formation 43. Later, Nian *et al.* observed that during osteoblast differentiation, levels of LDHA, lactate, and H3K18la progressively increased. Elevated H3K18la in the JunB promoter region of osteoblasts enhances its transcription, thereby promoting osteoblast differentiation 62. Wu *et al.* reported that histone Kla and the expression of BMSC-related osteogenic genes are downregulated in osteoporosis patients. Enhanced glycolysis in endothelial cells can upregulate osteogenic-related genes through H3K18la, facilitating the differentiation of bone marrow mesenchymal stem cells into osteoblasts 63. Additionally, Hao *et al.* found that the bone morphogenetic protein (BMP) signaling pathway in cranial neural crest cells (CNCC) is essential for producing glycolytic lactate and subsequent histone Kla, influencing craniofacial morphology development 64.

Lactylation is crucial for maintaining muscle cell homeostasis and promoting myogenesis. Lin *et al.* demonstrated that lactic acid produced during intense exercise acts as a signaling molecule, mediating Vps34 Kla to enhance autophagy in muscle tissue and maintain homeostasis. Moreover, autophagy, a conserved mechanism of cellular stability, promotes catabolism during exercise and clears damaged organelles and misfolded proteins, protecting skeletal muscles 21. Huang *et al.* found that Kla protein levels in skeletal muscle and liver peak 24 hours after high-intensity interval training and stabilize within 72 hours 65. Dai *et al.* confirmed that lactic acid enhances muscle generation by upregulating Neu2 expression via H3K9la activation 66. Zhou *et al.* indicated that blocking lactic acid production or uptake impairs myocyte differentiation 67. Desgeorges *et al.* discovered that histone Kla dynamics during muscle regeneration are critical for macrophage function. In macrophages, histone Kla predicts gene expression changes during ischemia-induced muscle regeneration 68. In summary, Kla is vital for various developmental processes.

### Others

Studies have shown that Kla is closely linked to DNA damage repair. For example, Sun *et al.* reported that Kla of PARP1 modulates its ADP-ribosylation activity, potentially aiding DNA repair 69. Chen *et al.* reported that MRE11 is lactylated at the K673 site by CBP acetyltransferase, increasing protein binding to DNA and promoting DNA end excision and homologous recombination (HR) 70. Chen *et al.* also found that lactate-driven lactation of NBS1 promotes HR-mediated DNA repair. NBS1 K388la is essential for forming the MRE11-RAD50-NBS1 complex and accumulating homologous recombination repair proteins at DNA double-strand breaks 20. However, this “protective umbrella” effect appears detrimental in tumor cells. Specifically, lactate facilitates DNA break repair in cancer cells, maintaining genomic stability and contributing to chemotherapy resistance 20, 69, 70. Disrupting the Kla process or inhibiting Kla modification with small-molecule polypeptides enhances tumor cell sensitivity to chemotherapy and improves drug efficacy.

Moreover, Zou *et al.* provided valuable insights into nonhistone Kla modifications for skin rejuvenation. The study showed that fibroblasts can take up extracellular lactate released by Poly-L-Lactic Acid via monocarboxylate transporter 1 (MCT1), which promotes Kla of lysine 752 on LTBP1 through a KAT8-dependent mechanism. This process increases type I and III collagen levels in fibroblasts, enhancing skin rejuvenation 71. Hu *et al.* found that lactate and lactylation levels increased after spinal cord injury. Lactate-mediated upregulation of H4K12la promotes PD-1 transcription in microglia, which facilitates microglial proliferation, scar formation, axon regeneration, and motor function recovery after spinal cord injury 72. This suggests that lactate and its mediated Kla play a crucial role in tissue repair via microglial activity, providing new therapeutic targets for spinal cord injury. Qiu *et al.* discovered that H3K18la regulates the transcriptional activation of the duox gene, leading to ROS production. ROS further promotes H3K18la, forming a positive feedback loop. This H3K18la-ROS-driven cycle contributes to light exposure-induced neutrophil recruitment in zebrafish 73. The study emphasized the role of Kla mediated by light-dark cycles in optimizing immune function.

## "Deleterious" regulation of lactylation in pathological processes

Kla is a double-edged sword for health, with elevated levels potentially causing pathogenic effects in diseases. This review will summarize Kla's roles and mechanisms in tumors and systemic non-tumor diseases, and evaluate its potential as a therapeutic target.

### Tumor and lactylation regulation

**Figure 4:**
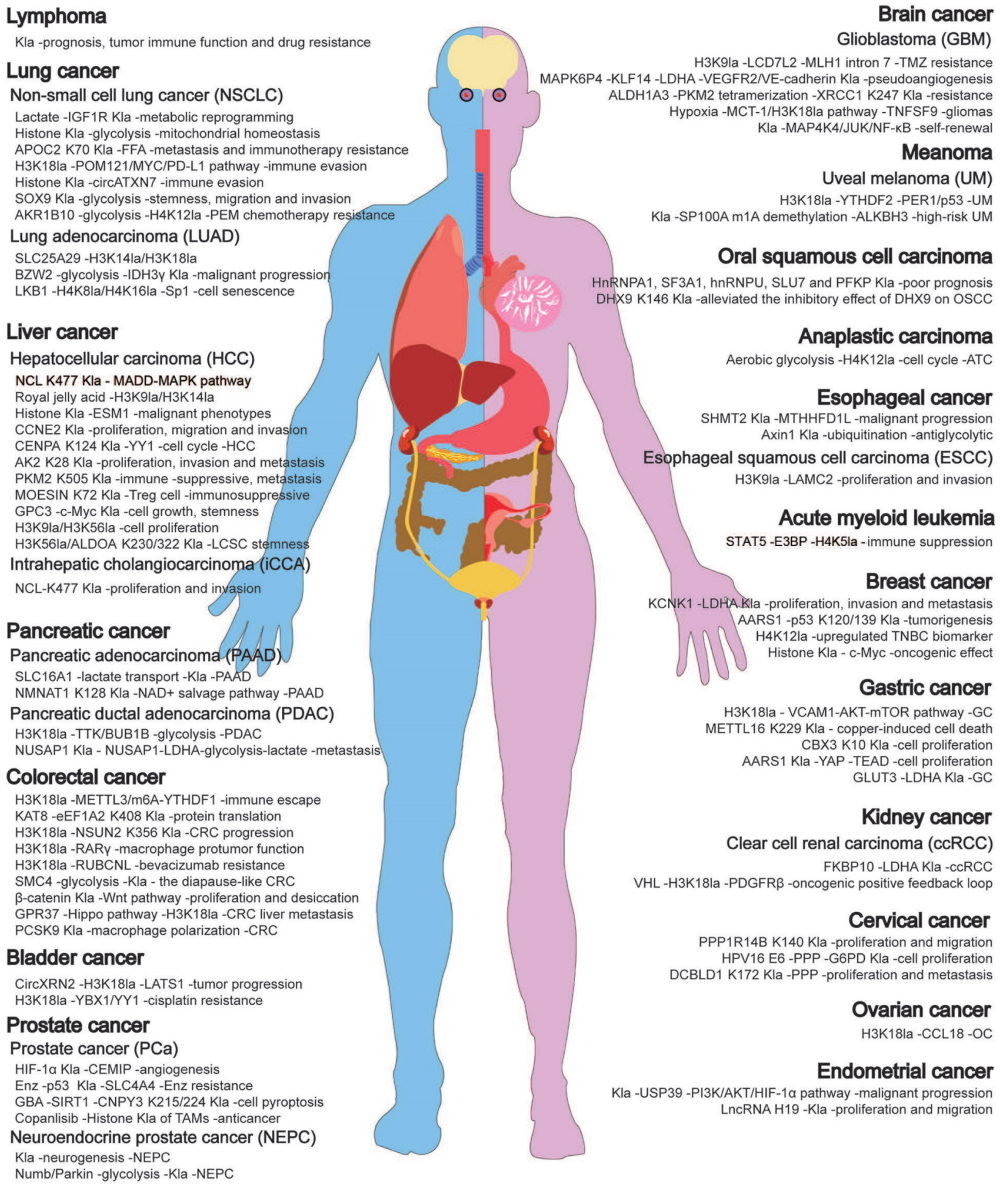
"Deleterious" regulation of lactylation in malignant tumors. Lactylation is widely present in various cancers and is closely related to the occurrence and progression of malignancies. It plays a crucial role in tumor metabolism, tumor microenvironment, tumor immunosuppression, tumor progression, metastasis, and treatment resistance through multiple mechanisms.

Kla is commonly detected in various cancers and is associated with tumor occurrence, progression, and treatment response (Fig. 4, Table 3). While its role may differ depending on tumor type, stage, and individual factors, elevated Kla levels are often indicative of poor prognosis 74. Recently, targeting lactate-lactylation and its associated metabolic pathways has emerged as a promising research direction in cancer treatment 74. Next, we will discuss the current research status and future prospects of Kla in various cancers based on the latest 2022 global cancer burden data, aiming to offer new insights for cancer treatment and facilitate its clinical translation.

### Lung cancer

Lung cancer (LC) is the most prevalent cancer worldwide and a major cause of cancer-related deaths 75. Zhang *et al.* reported that IGF1R Kla is associated with lung cancer progression, as lactate-induced IGF1R Kla drives cell proliferation and metabolic reprogramming 75. Non-small cell lung cancer (NSCLC) accounts for 80-85% of LC cases. Jiang *et al.* demonstrated that lactate is crucial for metabolic dysregulation in NSCLC. Lactate-mediated Kla downregulated glycolytic enzymes (HK-1, PKM) and upregulated TCA cycle enzymes (SDHA, IDH3G), reducing glycolysis and maintaining mitochondrial homeostasis in NSCLC cells 76. Chen *et al.* revealed that lactate accumulation in NSCLC cells induces APOC2-K70 Kla, promoting extracellular lipolysis to produce FFA, enhancing metastasis, and contributing to immunotherapy resistance. Notably, the anti-APOC2-K70-lac antibody enhances tumor immunotherapy, suggesting potential for combinational approaches 19. Zhang *et al.* reported elevated pan-Kla and H3K18la levels in NSCLC tissues, which were positively associated with poor patient prognosis 77. H3K18la enhances immune evasion in NSCLC cells by activating the POM121/MYC/PD-L1 pathway 77. Inhibiting glycolysis with 2-DG and oxalate, or silencing LDHA and LDHB, lowered H3K18la levels and reduced immune evasion in NSCLC cells by enhancing CD8+ T cell cytotoxicity 77. Yan *et al.* found that hypoxia enhances sphere formation, migration, invasion, glucose consumption, lactate production, glycolysis, and global Kla. Hypoxia-induced SOX9 Kla promotes glycolysis, enhancing stemness, migration, and invasion in NSCLC cells 78. These findings suggest that targeting hypoxia could be an effective therapeutic strategy for NSCLC. Additionally, KRAS gene mutations are common oncogenic drivers in NSCLC. Zhou *et al.* demonstrated that lactate-induced histone Kla from KRAS-mutated tumor cells activates circATXN7 transcription, driving tumor immune evasion by increasing activation-induced cell death sensitivity in tumor-specific T cells 79.

Lung adenocarcinoma (LUAD) is the most common subtype of NSCLC, representing about 40% of lung cancer cases, with a poor prognosis and a 5-year survival rate of only 4-17% 80. Zheng *et al.* found that SLC25A29 expression correlates with lactate levels, and that H3K14la and H3K18la modifications play key regulatory roles in the SLC25A29 promoter 80. Wang *et al.* discovered that BZW2 promotes LUAD progression by enhancing lactate production through glycolysis and Kla of IDH3G. Inhibiting Kla suppresses LUAD progression, and combining BZW2 knockdown with 2-DG treatment significantly inhibits tumor growth in mice 81. Liu *et al.* found that LKB1 suppresses Kla of histones H4 (Lys8) and H4 (Lys16), alters Sp1 activity, inhibits telomerase, and promotes senescence in LUAD cells 82. In conclusion, these studies offer new possibilities for LUAD treatment and support targeting Kla in LUAD therapy.

Additionally, brain metastasis (BM) is a malignant event and a key factor in the poor prognosis of NSCLC patients 83. Pemetrexed (PEM), a first-line chemotherapy drug capable of crossing the blood-brain barrier, faces limitations in treating lung cancer brain metastases due to drug resistance 83. Wang *et al.* reported that AKR1B10 promotes glycolysis by upregulating LDHA expression and increasing lactate levels. This leads to H4K12la, which activates CCNB1 transcription, accelerates DNA replication, and drives the cell cycle, ultimately contributing to acquired PEM resistance in lung cancer bone marrow 83.

### Colorectal cancer

Colorectal cancer (CRC) is the second leading cause of death worldwide and the third most common cancer 84. Huang *et al.* found significantly elevated pan-Kla levels in CRC, especially in malignant tumors, suggesting that pan-Kla may serve as an independent prognostic factor for CRC. This suggests that risk models based on Kla-related genes could significantly improve the management and treatment outcomes of CRC patients 84. Xiong *et al.* found that lactate accumulation in the tumor microenvironment (TME) enhances the transcription of methyltransferase METTL3 in tumor-infiltrating macrophages (TIMs) through H3K18la. METTL3 further mediates m6A modification of JAK1 mRNA, and the m6A-YTHDF1 axis ultimately promotes JAK1 protein translation and STAT3 phosphorylation, facilitating CRC immune evasion and tumor progression 47. Xie *et al.* reported that KAT8 promotes CRC development by lactylating the lysine 408 site of eEF1A2, enhancing protein translation efficiency 22. Chen *et al.* found that lactate accumulation in CRC cells activates NSUN2 transcription via H3K18la and induces NSUN2 K356la, promoting CRC progression 85. Li *et al.* reported that tumor-derived lactate in CRC enhances H3K18la, suppresses RARγ transcription, elevates IL-6 levels in the TME, and activates STAT3 signaling, endowing macrophages with pro-tumor functions 86. Li *et al.* also showed that tumor-derived lactate promotes RUBCNL expression via H3K18la in CRC, exacerbating resistance to bevacizumab therapy 87. Sun *et al.* revealed that SMC4 downregulation induces abnormal glycolysis, lactate accumulation, and histone Kla, leading to increased ABC transporter expression and a dormancy-like CRC cell phenotype with low proliferation and chemoresistance 88. Miao *et al.* found that hypoxia-induced β-catenin Kla promotes CRC cell proliferation and stemness via the Wnt pathway, exacerbating malignant behaviors 89. Zhou *et al.* identified that GPR37 activates the Hippo pathway, upregulates LDHA expression and glycolysis, increases H3K18la levels, and enhances CXCL1 and CXCL5 expression, promoting CRC liver metastasis 90.

Given Kla's multifaceted roles in CRC, targeting Kla offers promising therapeutic potential. For example, Wang *et al.* found that silencing PCSK9 reduced levels of lactate, protein Kla, and macrophage migration inhibitory factor, promoting M1 macrophage polarization while inhibiting M2 polarization, ultimately suppressing CRC progression 91. Similarly, Li *et al.* observed elevated histone Kla in CRC patients resistant to bevacizumab. Under hypoxic conditions, inhibiting histone Kla effectively suppressed CRC tumor formation, progression, and survival 87. Combining drugs that inhibit lactylation and autophagy enhanced the efficacy of bevacizumab in CRC treatment 87. Furthermore, Gu *et al.* showed that *Escherichia coli* could inhibit NF-κB recruitment to the NLRP3 promoter through RIG-I Kla in macrophages, affecting the immunosuppressive activity of Tregs and the antitumor function of CD8+ T cells 92. These findings suggest that the tumor-resident microbiome may be a potential target for preventing and treating colorectal liver metastases. Collectively, these studies indicate that targeting the Kla process could provide new therapeutic strategies for CRC prevention and treatment.

### Liver cancer

Hepatocellular carcinoma (HCC) is a common liver cancer closely linked to metabolic processes. Recent studies have identified widespread Kla modifications in HCC, affecting enzymes in various pathways, with Kla levels correlating to HCC aggressiveness and mutations 93. Yang *et al.* were the first to map the landscape of Kla modifications in HCC. Through integrated lactyl-proteomic and proteomic analyses of tumor and adjacent liver tissues, they revealed that Kla is a widespread modification extending beyond histones and transcriptional regulation 93. Notably, their analysis of Kla-modified substrates demonstrated significant impacts on enzymes in key metabolic pathways, including glycolysis, the TCA cycle, amino acid metabolism, fatty acid metabolism, and nucleotide metabolism. Higher Kla levels on these pathway proteins were closely associated with invasive clinical features and driver mutations in HCC 93. Subsequently, Kla has been extensively studied in HCC. Cheng *et al.* developed an effective prognostic model and identified lactylation-related genes (LRGs) associated with HCC prognosis. They discovered that patients with low-risk LRG scores responded better to most targeted drugs and immunotherapies, while those with high-risk scores were more sensitive to chemotherapy and sorafenib, suggesting that LRG markers could serve as biomarkers for effective clinical treatment of HCC 94. Jin *et al.* further confirmed that histone Kla in liver cancer is closely associated with tumor progression, lymph node metastasis, and staging. Collectively, these findings suggest that Kla may serve as a diagnostic and prognostic biomarker for HCC and that targeting lactate immunometabolism and Kla could offer a potential therapeutic strategy 27.

In recent years, increasing evidence has shown that Kla promotes the progression of HCC. For instance, Zhao *et al.* found that histone Kla levels, particularly H3K9la and H3K56la, are significantly elevated in HCC tissues and cells. This modification enhances the malignant phenotype, tumor growth, and metastasis of HCC cells by upregulating ESM1 expression 95. Jin *et al.* discovered that nonhistone Kla of CCNE2 promotes the proliferation, migration, and invasion of liver cancer cells. In contrast, the NAD-dependent deacetylase SIRT3 removes Kla from CCNE2, thereby regulating the cell cycle and inhibiting HCC progression 27. The study also suggested that andrographolide enhances SIRT3-mediated deacetylation of CCNE2, boosting its anti-HCC effect 27. Liao *et al.* reported that centromere protein A (CENPA) can be lactylated at K124, which activates CENPA. This activation, in turn, drives the expression of CCND1 and NRP2, promoting HCC progression 96. Yang *et al.* found that HCCpatients with the proliferative subtype had higher levels of AK2 Kla in tumor tissues, which was associated with poor prognosis. AK2 K28la inhibited its kinase activity, disrupting intracellular energy balance and promoting HCC cell proliferation, invasion, and metastasis 97. Qian *et al.* observed that under high-glucose conditions, glycolysis in HCC cells increased, leading to elevated lactate levels. This, in turn, promoted PKM2 K505 Kla, inhibiting FBP binding to PKM2 and facilitating its transition from a tetramer to a dimer. PKM2 Kla also enhanced its shift from glycolytic function to gene transcription regulation, which reinforced the immunosuppressive microenvironment and promoted HCC metastasis 98. Gu *et al.* found that effective anti-PD-1 treatment in HCC patients correlated with lower levels of MOESIN Kla in Treg cells compared to non-responders. The study showed that lactate regulated Treg cells through MOESIN K72la, enhancing the interaction between MOESIN, TGF-β receptor I, and downstream SMAD3 signaling. This process contributed to immunosuppression and facilitated HCC progression 99. Cai *et al.* identified the SRSF10/MYB/glycolysis/lactate axis as a key mechanism in immune evasion and resistance to anti-PD-1 therapy. SRSF10 upregulated lactate production, creating a positive feedback loop that enhanced glycolysis and H3K18la in tumor cells. Increased lactate levels promoted macrophage polarization to the M2 phenotype, suppressing CD8+ T cell activity. These findings suggest that the SRSF10 inhibitor 1C8 could overcome HCC resistance to anti-PD-1 therapy 100. Yao *et al.* found that knocking down GPC3 reduced overall Kla levels and c-myc Kla under hypoxic conditions, inhibiting HCC cell growth, stemness, and glycolysis. This suggests that GPC3-mediated Kla could be a promising therapeutic target for liver cancer 101. Pan *et al.* showed that lactate induced histone H3K9 and H3K56 lactylation and increased the expression of cell cycle-related proteins in HCC stem cells, stimulating cell proliferation and promoting HCC progression 102. Moreover, Feng *et al.* reported enhanced glycolytic metabolism, lactate accumulation, and elevated Kla levels in liver cancer stem cells (LCSCs) compared to HCC cells. H3K56la was closely related to tumorigenesis and LCSC stemness. Lactylation at ALDOA K230/322 played a crucial role in promoting LCSC stemness 103. This research underscores the importance of Kla in regulating LCSC stemness and its impact on HCC progression, suggesting that targeting LCSC lactylation may offer a promising therapeutic strategy for HCC 103.

Intrahepatic cholangiocarcinoma (iCCA) is a highly aggressive malignant liver tumor 104. Yang *et al.* reported that under hyperactive glycolysis, nucleolin (NCL) is primarily lactylated at lysine 477 by the acyltransferase P300, promoting iCCA cell proliferation and invasion. Further research showed that lactylated NCL binds to the primary transcript of MAP kinase-activating death domain protein (MADD) and facilitates efficient MADD translation by preventing premature stop codons through selective splicing. NCL Kla, MADD expression, and subsequent ERK activation drive xenograft tumor growth and correlate with overall survival in iCCA patients 104.

Additionally, studies suggest that certain natural compounds can inhibit lactate production and histone Kla, exerting anti-HCC effects. For example, demethylzeylone (DML) suppresses liver cancer stem cell tumorigenesis by inhibiting H3K9la and H3K56la 102. Similarly, royal jelly acid disrupts lactate production and specifically inhibits Kla at H3K9 and K14, reducing liver cancer cell proliferation and metastasis 105. Collectively, these studies highlight the role of Kla in HCC progression and propose that targeting Kla could provide novel therapeutic strategies for HCC.

### Breast cancer

Breast cancer is the most common malignancy and a leading cause of death among female cancer patients 106. Cui *et al.* found that H4K12la is significantly upregulated in triple-negative breast cancer (TNBC), serving as a novel biomarker 106. Li *et al.* discovered that lactate-induced H4K12la specifically suppresses SLFN5 expression, thereby promoting TNBC progression, and proposed a key lactylation-dependent oncogenic pathway 107. Hou *et al.* found that potassium two-pore domain channel subfamily K member 1 (KCNK1) accelerates glycolysis and lactate production by binding and activating LDHA. This process promotes Kla and induces the expression of downstream targets, including LDHA itself, leading to breast cancer proliferation, invasion, and metastasis 108. Zong *et al.* revealed that AARS1 acts as a lactate sensor mediating global Kla in breast cancer cells. AARS1 catalyzes lactylation at K120 and K139 in the DNA binding domain of p53, suppressing its phase separation, DNA binding, and transcriptional activation, thereby driving breast cancer development 109. Notably, this study employed an orthogonal translation system with pyrrolysyl-tRNA synthetase to enhance p53 Kla at specific sites, rather than adding exogenous lactate or overexpressing LDHA. The research also suggested using β-alanine to inhibit p53 Kla and restore its tumor-suppressor function, as β-alanine competes with lactate to bind AARS1, boosting chemotherapy effectiveness 109. This groundbreaking study not only unveiled a novel regulatory mechanism linking the metabolite lactate to p53 function but also opened new avenues for cancer treatment strategies 109.

Additionally, Madhura *et al.* showed that lactate, a tumor metabolite, promotes breast cancer progression by regulating histone Kla-dependent c-Myc expression 110. Liu *et al.* also found that asiatic acid enhances caspase-3 activity, inducing mitochondrial apoptosis, while modulating lactylation and 2-hydroxyisobutyrylation to inhibit breast cancer progression 111. In summary, these studies suggest that lactylation and its related pathways could provide new therapeutic targets for breast cancer.

### Gastric cancer

Gastric cancer (GC) is a malignant tumor from gastric mucosal cells, with high incidence and mortality, threatening human health 112. Studies reveal that histone Kla is elevated in GC tissues compared to adjacent non-cancerous tissues and correlates with poor prognosis, suggesting its potential as a prognostic biomarker 112. Moreover, bioinformatics analysis by Yang *et al.* identified six lactylation-related genes associated with GC prognosis, showing that lactylation scores are strongly correlated with overall survival and disease progression. Notably, patients with higher lactylation scores exhibit greater immune evasion and reduced response to immunotherapy 113. This implies that lowering lactylation levels to improve tumor sensitivity to ICIs could be a potential therapeutic strategy.

In terms of molecular mechanisms, Yang *et al.* discovered that in gastric cancer, glucose transporter 3 (GLUT3) promotes lactylation modification by regulating LDHA. Knockdown of GLUT3 significantly reduced the levels of LDHA, L-lactyl, H3K9, H3K18, and H3K56 114. Zhao *et al.* found that lactate in the TME promotes dynamic H3K18la in gastric cancer cells, upregulating VCAM1 transcription. This activates the AKT-mTOR signaling pathway, promoting tumor cell proliferation, epithelial-mesenchymal transition (EMT), and tumor metastasis. Furthermore, VCAM1 upregulates CXCL1 expression via the AKT-mTOR pathway, facilitating the recruitment of hGC-MSCs and M2 macrophages 115. Duan *et al.* revealed that chromobox protein homolog 3 (CBX3) K10la is significantly upregulated in various gastrointestinal tumors, including gastric cancer. This modification is closely linked to tumor cell proliferation and growth. Knockdown of CBX3 or blocking K10 Kla significantly inhibited tumor growth 116. Sun *et al.* found that copper ions regulate METTL16-K229 Kla via lactylases and the delactylase SIRT2, modulating its activity. Lactylated METTL16 induces methylation at the FDX1 mRNA-602 site, promoting FDX1 expression and leading to ferroptosis. SIRT2 inhibits METTL16 Kla, and combining the SIRT2 inhibitor AGK2 with the ferroptosis inducer Eles significantly enhances the treatment of malignant gastric cancer, especially for mucinous adenocarcinomas with high copper content 117. Ju *et al.* identified that AARS1 senses intracellular lactate and translocates to the nucleus, activating the YAP-TEAD complex and forming a positive feedback loop that promotes gastric cancer progression 32. Given the competitive binding of lactate and alanine to AARS1, this explains why high lactate levels do not inhibit general mRNA translation, which is essential for cell proliferation and tumor growth. Moreover, AARS1 Kla may serve as an alternative regulatory mechanism for YAP activity, independent of the canonical Hippo pathway. Li *et al.* found that lactate accumulation leads to H3K18la, which is enriched in the PD-L1 promoter region, thereby promoting PD-L1 transcription. This suggests that cancer-associated fibroblasts (CAFs) may reduce the efficacy of PD-1/PD-L1 blockade immunotherapy through glycolysis and lactate accumulation induced by LOX 118. In summary, lactylation plays a critical role in the development of gastric cancer. These findings not only expand the proteomic data on lactylation in gastric cancer but also suggest that lactylation could serve as a potential prognostic marker and a novel therapeutic target for gastric cancer.

### Pancreatic cancer

Pancreatic cancer (PAAD) is known for its aggressiveness, high mortality, and poor prognosis 119. Peng *et al.* identified 10 LRGs that are differentially expressed and have prognostic value, using RNA sequencing and clinical data analysis. These genes, including SLC16A1, HLA-DRB1, KCNN4, KIF23, and HPDL, were found to be strongly associated with overall survival in PAAD patients 119. Furthermore, the research confirmed that LRG-SLC16A1 regulates lactylation in pancreatic cancer cells by facilitating lactate transport. Reducing SLC16A1 and its lactylation significantly slows tumor progression, suggesting that targeting the SLC16A1/Kla pathway could be a potential therapeutic approach for PAAD 119. Huang *et al.* observed that the abundance of Pan-Kla is notably increased in PAAD patients and correlates with poor prognosis. The study also identified that lysine residue 128 of NMNAT1 plays a critical role in catalyzing lactylation. Lactylation of NMNAT1 enhances its nuclear localization and preserves its enzymatic activity, thus supporting the NAD+ salvage pathway and promoting tumor growth in PAAD 120. Zhao *et al.* discovered that RHOF promotes the Kla of Snail1 by enhancing PKM2-mediated glycolysis, which drives EMT in pancreatic cancer cells 121.

Pancreatic ductal adenocarcinoma (PDAC) presents significant challenges, with a 5-year survival rate of approximately 9% 122. Li *et al.* reported that histone Kla, especially H3K18la, is significantly increased in PDAC and associated with poor outcomes. H3K18la accumulates in promoter regions, activating transcription of mitotic checkpoint regulators TTK and BUB1B, which in turn increases P300 expression, intensifying PDAC glycolysis and dysfunction 122. Chen *et al.* discovered that lactate inhibits the degradation of nucleolar and spindle-associated protein 1 (NUSAP1) through Kla modification, upregulating NUSAP1 expression and forming a feedback loop that accelerates PDAC metastasis 123. Furthermore, Takata *et al.* proposed that Kla may play a role in the development of pancreatic epithelial tumors and could be a potential therapeutic target. Elevated Kla levels were observed in the nuclei of intraductal papillary mucinous neoplasms, non-invasive intraductal papillary mucinous carcinomas, and invasive cancers, along with increased hypoxia-inducible factor-1α levels, suggesting that hypoxia-related nuclear protein Kla could serve as a biochemical marker for pancreatic epithelial tumors 124. Overall, these studies underscore the pivotal role of Kla in pancreatic cancer progression and highlight potential targets for new therapeutic approaches.

### Esophageal cancer

Esophageal cancer (EC) is a prevalent and lethal gastrointestinal tumor. Recent research identifies the hypoxic microenvironment as a major driver of its rapid progression 125. Hypoxia not only accelerates tumor growth but also causes lactic acid buildup, which induces histone lysine Kla, influencing gene transcription and regulation. For example, Qiao *et al.* revealed that hypoxia-induced Kla of SHMT2 enhances MTHFD1L expression, promoting malignant progression in EC cells 125. Li *et al.* demonstrated that hypoxia increases Axin1 Kla, leading to its ubiquitination and degradation, which facilitates glycolysis and stemness in EC cells 126. Zang *et al.* found that hypoxia elevates histone H3K9la levels, boosting LAMC2 transcription and driving proliferation and invasion in esophageal squamous cell carcinoma 127. Additionally, Fu *et al.* reported that the long non-coding RNA AP001885.4 enhances esophageal squamous cancer cell proliferation through histone Kla, NF-κB transcriptional activation, and METTL3-mediated stabilization of c-Myc mRNA 128. These findings provide new insights into EC pathogenesis and suggest targeting histone Kla as a potential therapeutic strategy.

### Prostate cancer

Prostate cancer (PCa) is one of the most common malignancies in adult men 129. Pan *et al.* showed that the LRG prognostic model effectively predicts disease-free survival and treatment response in PCa patients 129. Luo *et al.* found that lactate enters PCa cells via MCT1, stabilizing HIF1α expression under normoxia through Kla, which promotes KIAA1199 transcription and angiogenesis 130. Recent studies indicate that regulating lactate metabolism and Kla can reverse chemotherapy resistance and enhance treatment efficacy. For instance, the androgen receptor inhibitor enzalutamide (Enz) is effective in advanced PCa 131, but long-term use may lead to resistance 132. Chen *et al.* found that long-term Enz treatment upregulates SLC4A4, which mediates P53 Kla via the NF-κB/STAT3/SLC4A4 axis, leading to Enz resistance and PCa progression 133. This suggests that targeting SLC4A4 could be a promising strategy for overcoming Enz resistance. Zhang *et al.* found that gambogic acid forms hydrogen bonds with Glu171 and Thr100 of CNPY3, recruiting the SIRT1 protein to bind with CNPY3. This inhibits lactylation at the K215 and K224 sites of CNPY3 and promotes the lysosome-dependent CatB/caspase 1/GSDMD pyroptotic pathway, inducing pyroptosis in DU145 cells and suppressing prostate cancer progression 26. These findings highlight the potential of inhibiting lactylation to induce cell pyroptosis and suppress tumor progression, offering strategies for developing innovative antitumor drugs. Kiranj Chaudagar *et al.* found that the PI3K inhibitor copanlisib reduced lactate production in tumor cells, inhibiting histone Kla in tumor-associated macrophages (TAMs) and enhancing their antitumor phagocytic activity. This activity was further enhanced by ADT/aPD-1 treatment but blocked by feedback activation of the Wnt/β-catenin pathway. This finding suggests an immunometabolic strategy combining lactate and PD-1-mediated TAM immunosuppression reversal with ADT treatment for patients with PTEN-deficient mCRPC 134. Furthermore, Chaudagar *et al.* observed that, compared to copanlisib monotherapy (37.5%), combining lactate inhibition in the TME and H3K18la inhibition in TAMs achieved an 80% overall response rate. This suggests that in PTEN/p53-deficient AVPC, disrupting lactate-mediated crosstalk between cancer cells and TAMs can achieve durable tumor control independent of ADT 135.

Neuroendocrine prostate cancer (NEPC) is a rare tumor, and increasing evidence suggests that it is induced by prostate cancer treatment drugs 136. Wang *et al.* revealed that during NEPC progression, prostate cancer cells enter an intermediate state marked by Zeb1 expression, characterized by EMT, stemness, and neuroendocrine traits. Zeb1 drives histone Kla, a process that influences NEPC development 136. Additionally, cellular plasticity and neuroendocrine differentiation in prostate cancer and lung adenocarcinoma contribute significantly to resistance against targeted therapies 137. He *et al.* identified the Numb/Parkin pathway as a key metabolic switch in cancer cell plasticity and a potential therapeutic target. Without this pathway, NEPC cells display numerous fragmented mitochondria with low membrane potential, leading to metabolic reprogramming 137. This reprogramming boosts glycolysis, producing lactate that enhances Kla and the transcription of neuroendocrine-related genes, accelerating NEPC progression 137.

### Cervical cancer

Cervical cancer is a prevalent gynecological malignancy among young women 138. He *et al.* observed that the expression of the lactate-related gene PPP1R14B negatively correlates with CD8+ T cell infiltration and is linked to lower survival rates. The K140R mutation in PPP1R14B reduces Kla levels in cervical cancer cells, promoting their proliferation and migration 138. Huang *et al.* reported that histone Kla driven by GPD2 induces M2 macrophage polarization, facilitating the malignant transformation and progression of cervical cancer 139. Meng *et al.* demonstrated that the HPV16 E6 protein enhances cervical cancer cell proliferation by activating the pentose phosphate pathway (PPP) and suppressing the Kla of G6PD dimers 140. In another study by Meng *et al.*, lactylation at K172 of Discoidin, CUB, and LCCL domain-containing protein 1 (DCBLD1) upregulates DCBLD1 expression, which in turn increases G6PD expression and enzymatic activity. This activation of the PPP promotes the proliferation and metastasis of cervical cancer cells 141. Overall, the research is limited, and the precise characterization and functional significance of the non-histone Kla in cervical cancer still require further exploration.

### Bladder cancer

Bladder cancer (BCa) is one of the most common malignant tumors in the male urinary system, with a poor prognosis 142. Xie *et al.* discovered that in BCa, circXRN2 (a negative regulator of glycolysis and lactate production) interacts with SPOP, inhibiting the ubiquitination and degradation of LATS1. This, in turn, activates the Hippo signaling pathway, which suppresses the transcription of lipocalin-2 by blocking H3K18la on its promoter. This process ultimately slows tumor growth and metastasis 142. Furthermore, studies indicate that BCa patients often develop resistance to platinum-based chemotherapy, especially cisplatin 143. Li *et al.* discovered that key transcription factors YBX1 and YY1, driven by H3K18la, contribute to cisplatin resistance in bladder cancer. Inhibiting H3K18la restores cisplatin sensitivity in resistant epithelial cells 143. Although research is still limited, these findings reveal new therapeutic targets, offering promising strategies for clinical intervention in human bladder cancer.

### Kidney cancer

Renal cell carcinoma is one of the three major malignant tumors of the urinary system, originating from the epithelial cells of the proximal renal tubules. Clear cell renal cell carcinoma (ccRCC) accounts for approximately 80% of renal cancer cases and is a metabolic disease driven by genetic mutations and epigenetic alterations 144. In recent years, studies have revealed that lactylation modifications play a crucial role in ccRCC and may serve as prognostic diagnostic markers 145. TIMP1, a key gene linked to histone Kla in ccRCC, holds substantial prognostic value, especially in a sex-dependent manner, either on its own or in combination with its receptor 144. FKBP10 plays a critical role in hypoxia and glycolytic pathways during ccRCC progression. It binds to LDHA, a major glycolytic regulator, through its C-terminal domain, enhancing LDHA-Y10 phosphorylation. This promotes the Warburg effect, histone Kla, and ccRCC progression while affecting sensitivity to HIF2α blockade 146. Additionally, mutations in the von Hippel-Lindau (VHL) tumor suppressor gene are common in ccRCC, resulting in metabolic changes and increased lactate production, creating a lactate-rich TME 147. Inactive VHL correlates with high levels of histone Kla, which is linked to poor prognosis 147. VHL inactivation activates H3K18la through an HIF-dependent mechanism, promoting PDGFRβ gene transcription. This PDGFRβ signaling further amplifies H3K18la, forming a carcinogenic feedback loop that worsens ccRCC 147. These findings suggest that inhibiting lactylation may offer a promising therapeutic strategy for ccRCC.

### Glioma

Gliomas are the most common and aggressive brain tumors, characterized by high proliferation, abnormal glycolysis, and poor prognosis. Glioblastoma (GBM) is the most malignant and treatment-resistant subtype 148. Zhang *et al.* found that the pseudogene MAPK6P4 encodes a functional peptide, P4-135aa, which phosphorylates KLF15 at the S238 site, enhancing its stability and nuclear entry to activate LDHA transcription. LDHA binds to VEGFR2 and VE-cadherin, promoting their Kla and facilitating glioblastoma vascular mimicry 148. Li *et al.* found that hypoxia-induced histone Kla activates NF-κB-related LINC01127 expression via the MAP4K4/JNK/NF-κB axis, promoting GBM cell self-renewal 149. Hypoxia is a key factor in poor prognosis and treatment challenges in gliomas 150. Li *et al.* found that hypoxia regulates TNFSF9 expression via the MCT-1/H3K18la pathway, inducing M2 macrophage polarization and promoting glioma progression 150. Li *et al.* also found that ALDH1A3-mediated PKM2 tetramerization promotes lactate accumulation in GBM stem cells, inducing XRCC1 K247 Kla, which confers treatment resistance. D34-919 disrupts the ALDH1A3-PKM2 interaction, enhancing GBM cell sensitivity to radio-chemotherapy 151. This suggests that ALDH1A3-mediated PKM2 tetramerization could be a potential therapeutic target to improve the response of ALDH1A3-high GBM to radio-chemotherapy 151. Wang *et al.* found that lactate from patient-derived glioma stem cells and microglia/macrophages induces epigenetic reprogramming of tumor cells via histone Kla regulated by CBX3, promoting an immunosuppressive transcriptional program. This upregulates CD47 (a “don't eat me” signal) in GBM cells, inhibiting phagocytosis and promoting immune evasion 152. This suggests that this mechanism can be leveraged to enhance immunotherapy efficacy.

Temozolomide (TMZ) resistance poses a significant challenge in GBM treatment 153. Yue *et al.* reported that Kla is upregulated in recurrent GBM tissues and TMZ-resistant cells, especially H3K9la. H3K9la induces TMZ resistance by LUC7L2-mediated retention of MLH1 intron 7 153. Additionally, Leo *et al.* showed that pERK-driven glucose metabolism enhances MDM immunosuppressive activity via histone Kla, promoting T-cell accumulation and slowing tumor growth. This process can be combined with immunotherapy to stop GBM progression 154. These findings provide new targets and strategies for GBM treatment.

### Uveal melanoma

Uveal melanoma (UM), the most common intraocular malignancy in adults, is highly aggressive and prone to metastasis 155. Yu *et al.* reported that histone pan-Kla is significantly elevated in UM tissues and cell lines, correlating with poor outcomes 155. H3K18la promotes overexpression of the m6A reader YTHDF2, which binds to m6A sites on tumor-suppressor mRNAs PER1 and TP53, leading to their degradation and driving UM progression 155. Targeting H3K18la can effectively block tumor growth, offering a promising new therapeutic approach. Gu *et al.* also found a strong positive correlation between UM and intracellular histone Kla. Kla increases ALKBH3 expression by demethylating m1A on SP100A, which decreases PML body formation and promotes UM malignancy 156. These findings offer new perspectives for treating UM.

### Other malignancies

The high global incidence and mortality rates of these cancers highlight the critical roles of lactate and Kla in tumor metabolism, microenvironment, immune suppression, growth, metastasis, and treatment resistance. For example, Song *et al.* identified 1,011 and 1,197 Kla sites in oral squamous cell carcinoma cells, respectively, observing enrichment in the spliceosome, ribosome, and glycolysis/gluconeogenesis pathways. Lactylation modifications were detected in spliceosomal proteins such as hnRNPA1, SF3A1, hnRNPU, and SLU7, as well as in the glycolytic enzyme PFKP. Furthermore, lactylation levels were negatively correlated with patient prognosis 157. Sun *et al.* found that LRGs effectively identify high-risk patients and predict outcomes in multiple myeloma 158. Zhu *et al.* reported elevated lactate levels in lymphoma patients, where Kla influences prognosis and immune function in diffuse large B-cell lymphoma 159. Similarly, Yu *et al.* linked LRGs to tumor classification and immunity in ovarian cancer (OC), predicting patient outcomes 160. Sun's research showed that lactate activates CCL18 expression in macrophages via H3K18la, promoting OC progression 161. Wei *et al.* observed significantly increased Kla in endometrial cancer tissues, where histone Kla enhances USP39 expression, driving the PI3K/AKT/HIF-1α pathway to promote malignancy 162. Treatment with 2-deoxy-D-glucose and sodium acetate reduced Kla levels, inhibiting cancer cell proliferation and migration 162. Wen *et al.* reported elevated aerobic glycolysis and histone Kla in endometriosis, with lncRNA H19 overexpression exacerbating metabolic abnormalities and promoting proliferation and migration 163. Huang *et al.* demonstrated that high STAT5 expression in acute myeloid leukemia leads to lactate accumulation, which promotes E3 ligase translocation and H4K5la, inducing PD-L1 transcription and immune suppression, suggesting the potential for PD-1-based immunotherapy 164. Wang *et al.* noted that FOXP3+ NKT-like cells utilize lactate via high MCT1 and lactate dehydrogenase B expression, maintaining immune suppression in malignant pleural effusions 165. Jia *et al.* found significantly higher lactate levels in lung and gastric cancer tissues compared to adjacent tissues, with lactate acting as a signaling molecule to enhance autophagy and tumor progression via KAT5/TIP60-mediated Kla at specific lysine residues 21. Wang *et al.* observed that anaplastic thyroid carcinoma (ATC) exhibits high histone Kla levels, with aerobic glycolysis increasing intracellular lactate utilization and disrupting cell cycle gene expression. Blocking Kla mechanisms, combined with BRAFV600E inhibitors, suppressed ATC progression 166. In summary, Kla significantly influences tumor epigenetics and gene expression, offering new therapeutic perspectives. Targeting tumor Kla, particularly in combination with immunotherapy, holds significant research potential.

### Nontumor-related diseases and lactylation regulation

**Figure 5:**
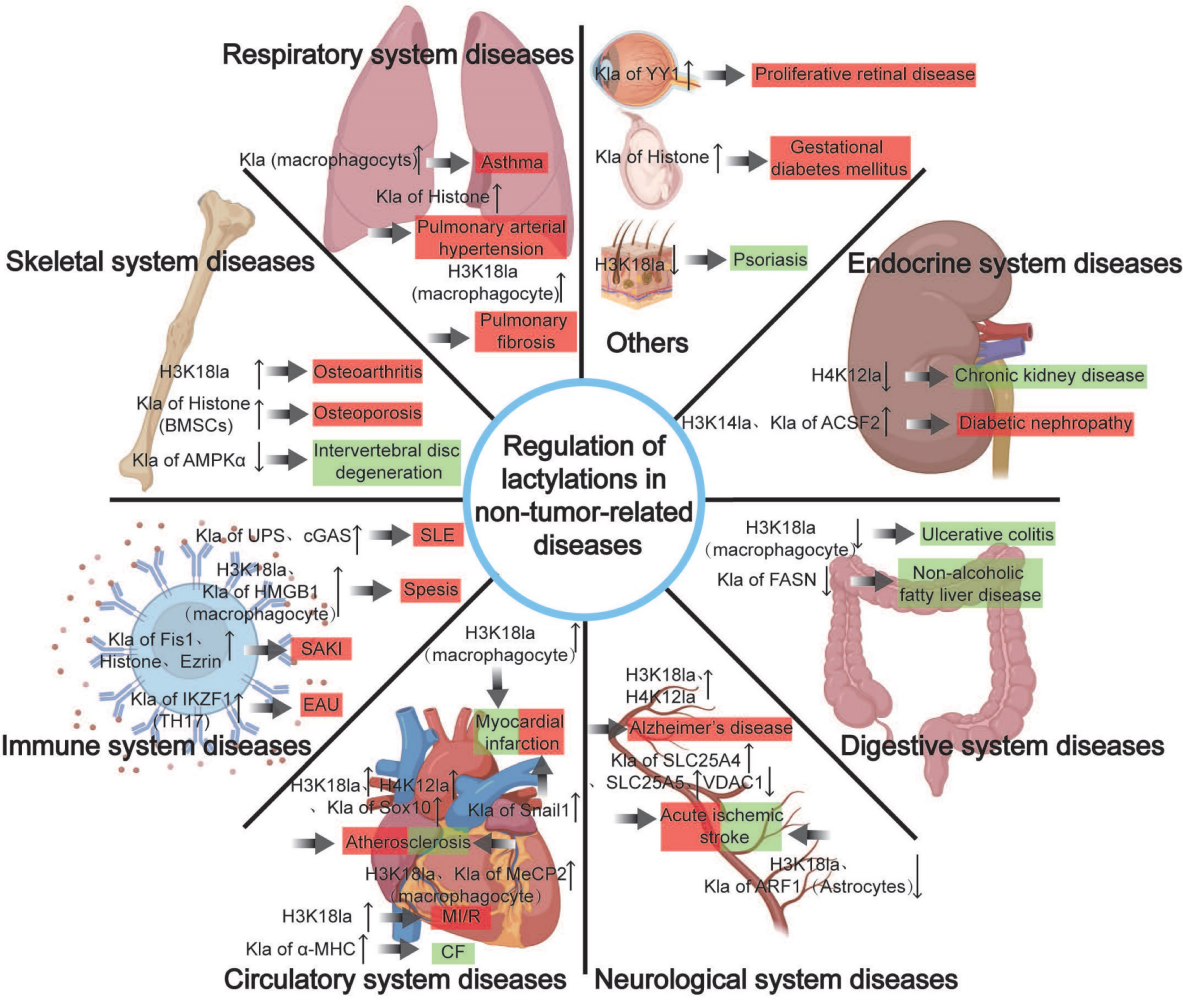
Lactylation in nontumor related diseases. Lactylation occurs in various non-tumor cells and is associated with numerous non-cancerous diseases. It significantly impacts the immune, respiratory, circulatory, nervous, skeletal, digestive, and endocrine systems, influencing the onset and regulation of these conditions. Note: Red indicates disease progression, while green signifies disease remission. Created in https://BioRender.com.

Recent evidence shows that Kla occurs in many nontumor cells and a variety of noncancerous diseases. We then classify and summarize the functions, molecular mechanisms, and potential applications of Kla in nontumor diseases (Fig. 5).

### Immune system diseases

Sepsis occurs when the host immune system cannot control infections, causing systemic inflammation and multiple organ failure 167. Chu *et al.* observed significantly elevated H3K18la levels in septic shock patients, with its expression positively correlated to APACHE II scores, mechanical ventilation duration, serum lactate, and procalcitonin levels 167. These findings suggest that H3K18la could be a biomarker for diagnosing and predicting septic shock severity, reflecting critical illness and infection 167. Yang *et al.* showed that serum lactate and high-mobility group box 1 (HMGB1) are positively linked to sepsis-related mortality. In sepsis, macrophage lactate promotes HMGB1 Kla via p300/CBP. Lactylated/acetylated HMGB1 is secreted through the exosome pathway, reducing VE-cadherin and claudin-5 levels, increasing ICAM1 levels, disrupting endothelial integrity, and increasing vascular permeability 39. This causes endothelial barrier dysfunction, accelerating sepsis progression 39. An *et al.* found that elevated lactate levels independently increase the risk of sepsis-associated acute kidney injury (SAKI). SIRT3 downregulation leads to hyperacetylation and inactivation of PDHA1, causing excessive lactate production. This, in turn, mediates Fis1 Kla, worsening SAKI. Lowering lactate levels and Fis1 secretion alleviates SAKI 168. Qiao *et al.* identified histone Kla as a contributor to renal impairment in SAKI. They found lactate-dependent histone modifications enriched at the RhoA promoter region. Histone Kla activates the RhoA/ROCK/Ezrin pathway, triggering NF-κB, inflammation, apoptosis, and kidney dysfunction. Multiple Kla sites were identified on Ezrin, with K263 Kla playing a key role in inflammatory metabolic adaptation in renal proximal tubular epithelial cells 169. Wu *et al.* found that METTL3, regulated by histone Kla, facilitates ferroptosis in sepsis-associated acute lung injury via m6A modification of ACSL4 170.

Systemic lupus erythematosus (SLE) is an autoimmune inflammatory connective tissue disease and a classic example of an “interferon type I disease 171. Zhang *et al.* found that mtDNA in SLE patients drives glycolysis to produce lactate, which promotes cGAS Kla. This process inhibits its binding to the E3 ubiquitin ligase MARCHF5, blocking cGAS degradation and triggering a strong IFN-I response 171. Developmental defects in erythrocytes (RBCs) have also been implicated as triggers for SLE 172. Caielli *et al.* reported that during RBC maturation, HIF activates the UPS and mediates UPS Kla, leading to an abnormal increase in mature RBCs. When engulfed by macrophages, mtDNA from these RBCs stimulates the cGAS/STING pathway, driving type I IFN production and SLE 173. Lupus nephritis (LN), a significant risk factor for SLE morbidity and mortality, has been linked to lactate metabolism. Sun *et al.* identified lactate-related biomarkers COQ2, COQ4, and NDUFV1 as being associated with LN, suggesting a role for lactate metabolism and protein Kla in its onset 174.

Experimental autoimmune uveoretinitis (EAU) is a T-cell-mediated, organ-specific autoimmune disease. Fan *et al.* demonstrated that lactylation from lactic acid regulates CD4+ T cell differentiation. Hyper-lactylation at the Lys164 site of Ikzf1 directly influences the expression of TH17-related genes, such as Runx1, Tlr4, IL-2, and IL-4, promoting TH17 differentiation. Suppressing lactylation inhibits TH17 cell differentiation and reduces EAU inflammation 175. These findings show how glycolysis-driven protein lactylation promotes TH17 differentiation, suggesting Ikzf1 Kla as a potential therapeutic target for autoimmune diseases.

Crohn's disease (CD) involves immune dysregulation in the gut, often linked to metabolic abnormalities. Wu *et al.* analyzed lactylation levels in immune cells using single-cell data and observed significant variations among cell types 176. Notably, Lactylation levels were higher in immune cells from inflamed areas. This highlights the close relationship between lactylation-related genes and inflammatory cell changes in CD patients.

### Respiratory system diseases

Pulmonary fibrosis is a chronic respiratory disease characterized by the gradual replacement of lung tissue with fibrotic tissue. Recent studies increasingly show that elevated lactate levels in human alveolar macrophages lead to increased histone Kla 177. Histone Kla induces high expression of pro-fibrotic-related genes, contributing to disease progression 177. For instance, Cui *et al.* found that lactate produced by myofibroblasts enriches H3K18la in the promoter regions of pro-fibrotic genes, such as ARG1, PDGFA, VEGFA, and THBS1, promoting their expression. This enhances the pro-fibrotic activity of alveolar macrophages, exacerbating pulmonary fibrosis 177. Similarly, Wang *et al.* reported that extracellular lactate from myofibroblasts increases overall Kla and H3K18la levels through MCT1. H3K18la facilitates the progression of arsenic-related idiopathic pulmonary fibrosis via the YTHDF1/m6A/NREP pathway 178.

Pulmonary arterial hypertension (PAH) is a severe condition caused by increased pulmonary vascular resistance and remodeling, ultimately leading to right heart failure or death. Lactate plays a critical role in PAH development by influencing disease progression through lactylation 179. Chen *et al.* discovered that hypoxia-induced mitochondrial reactive oxygen species (mROS) inhibit the hydroxylation of HIF-1α, triggering a glycolytic shift in pulmonary arterial smooth muscle cells (PASMCs) via the upregulation of the HIF-1α/PDK1&2/p-PDH-E1α axis, resulting in lactate accumulation and histone Kla 179. Enhanced Kla of HIF-1α targets, such as Bmp5, Trpc5, and Kit, promotes PASMC proliferation. PDK1&2 knockout reduced lactate production, histone Kla, and PASMC proliferation. Additionally, pharmacological inhibition of lactate dehydrogenase reduced histone Kla, improved PASMC proliferation, and alleviated vascular remodeling in hypoxia-induced PH rat 179. These findings highlight the potential of targeting lactate as a therapeutic strategy against vascular remodeling.

Asthma, a chronic lung disease caused by airway inflammation and constriction, leads to difficulty in breathing 180. Dexamethasone has been shown to exert anti-asthmatic effects by modulating the HIF-1α-glycolysis-lactate axis and protein Kla, offering a new therapeutic approach for eosinophilic asthma 180. In summary, lactate and histone Kla play crucial roles in the pathogenesis of various respiratory diseases, providing new strategies for their treatment.

### Circulatory system diseases

Cardiovascular diseases (CVDs), including myocardial infarction (MI), heart failure, atrial fibrillation, and atherosclerosis, pose a major global health threat 181. MI remains one of the leading causes of death and disability worldwide 181. Post-MI, lactate exacerbates heart dysfunction and fibrosis by inducing Snail1 Kla and activating the TGF-β/Smad 2 pathway, which drives endothelial-to-mesenchymal transition 181. Moreover, timely activation of reparative signals in monocytes and macrophages is essential to mitigate early inflammatory damage and facilitate repair 181. Wang *et al.* found that treating MI-affected mice with sodium lactate increased H3K18la in circulating monocytes and infiltrating macrophages, promoting angiogenesis and improving cardiac dysfunction 35. The study further highlighted that metabolic reprogramming and MCT1-mediated lactate transport enhance histone Kla, with GCN5 serving as a lactylation writer to regulate gene transcription. These findings provide new insights into the metabolic-epigenomic-immune mechanisms after MI, offering theoretical foundations and potential applications for improving cardiac repair with significant clinical value 35.

Myocardial ischemia/reperfusion (I/R) injury significantly contributes to adverse outcomes post-MI 182. Xu *et al.* reported that lactate affects cardiomyocyte size and apoptosis under OGD/R by elevating protein Kla levels and enhancing the intracellular YTHDF2, which subsequently upregulates Ras GTPase-activating protein-binding protein 1 (G3BP1) 182. Targeting Kla or inhibiting YTHDF2/G3BP1 may help alleviate acute injury and pathological remodeling caused by myocardial I/R, offering a potential therapeutic approach for heart diseases 182. Yao *et al.* found that increased LDHA activity raises lactate levels, promotes H3K18la, upregulates HMGB1 expression, induces apoptosis, and worsens cerebral I/R injury 183. Du *et al.* demonstrated that HSPA12A protects the liver from I/R injury by reducing HMGB1 Kla and secretion through glycolysis inhibition, thereby suppressing macrophage chemotaxis and inflammation 184. Interestingly, some studies suggest maintaining lactylation could mitigate MI/R injury. Yu *et al.* observed that HSPA12A stabilizes HIF1α protein via SMURF1, enhancing glycolytic gene expression, sustaining aerobic glycolysis, preserving H3 lactylation, and improving cardiomyocyte survival, thus reducing MI/R injury 185. Further research is needed to clarify the role and mechanisms of lactylation in I/R-induced diseases.

Atherosclerosis (AS) is a leading cause of CVD. Research indicates that elevated Kla levels exacerbate AS progression. For example, Dong *et al.* found increased aerobic glycolysis and lactate levels in endothelial cells within atherosclerotic regions 186. ASF1A-dependent p300-mediated H3K18la promotes AS by regulating endothelial-to-mesenchymal transition (EndMT). Pharmacological inhibition and advanced PROTAC suppression of glycolysis can reduce H3K18la, SNAI1 transcription, and EndMT-induced AS 186. Vascular smooth muscle cell (VSMC) senescence, marked by metabolic abnormalities, is another critical factor in AS development 187. Li *et al.* discovered that TRAP1-mediated metabolic reprogramming promotes H4K12la by downregulating HDAC3. H4K12la enriches senescence-associated secretory phenotype (SASP) gene promoters, activating their transcription, accelerating VSMC senescence, and driving AS 187. Xu *et al.* showed that sustained inflammatory damage triggers TNF-α-induced Sox10 Kla via the PI3K/AKT pathway, leading to VSMC transdifferentiation. This results in macrophage-like VSMC accumulation, vascular inflammation, pyroptosis-driven hyperplasia, and AS-related complications 185. Interestingly, some studies suggest enhancing lactylation may reduce AS risk. Zhang *et al.* found that H3K18la mediated by MCT4, associated with lactate efflux, activates repair genes and prevents AS. Inhibiting macrophage MCT4 promotes repair mechanisms 188. Chen *et al.* demonstrated that exercise induces MeCP2 K271 lactylation in macrophages within aortic root plaques. MeCP2 K271la-H3K36me3/RUNX1 promotes pro-repair M2 macrophage polarization, reducing plaque size, necrotic core area, and lipid deposition while increasing collagen content and suppressing AS 189. This highlights that interventions enhancing MeCP2 K271 lactylation could promote plaque stability by increasing pro-repair M2 macrophage infiltration, thereby lowering the risk of atherosclerotic CVD 189. The study also identified RUNX1 as a potential therapeutic target in exercise therapy, offering insights for new target discovery. In summary, the dual role of lactate in AS progression warrants further investigation.

Medial arterial calcification, common in chronic kidney disease and diabetes. Ma *et al.* discovered NR4A3 is a key regulator in the progression of apolipoprotein A-IV-induced atherosclerosis. NR4A3-mediated histone Kla represents a novel metabolome-epigenome signaling cascade involved in the development of medial arterial calcification 190. Huang *et al.* demonstrated that lumican mediates H3K14la and H3K9la, thereby promoting aortic valve calcification and suggesting that lumican could be a potential therapeutic target for calcific aortic valve disease (CAVD) 191. Wang *et al.* found that andrographolide (AGP) reduces CAVD by regulating LDHA to disrupt lactate production and inhibit VIC calcification. Additionally, the study identified p300 acetyltransferase as the molecular target of AGP in suppressing H3Kla. The suppression of H3Kla and H3K9la by AGP was associated with decreased Runx2 expression 192.

Moreover, Zhang *et al.* found that α-myosin heavy chain (α-MHC) undergoes lactylation at lysine 1897 (K1897). When K1897 lactylation is absent, the interaction between α-MHC and titin is reduced, impairing cardiac structure and function 28. The study identified p300 and Sirtuin 1 as the lactyltransferase and delactylase for α-MHC, respectively. It also showed that reducing lactate production, either chemically or genetically, lowers α-MHC Kla, weakens the α-MHC-titin interaction, and worsens heart failure. On the other hand, increasing lactate levels by administering sodium lactate or inhibiting key lactate transporters in cardiomyocytes enhances K1897 Kla and strengthens the α-MHC-titin interaction, improving heart failure outcomes 28. In conclusion, lactate and its role in Kla are increasingly recognized in cardiovascular diseases. Targeting lactate production, modulating its transport, and regulating circulating lactate levels could offer promising therapeutic strategies for heart disease.

### Neurological system diseases

Lactylation in the nervous system may hold broad biological significance, varying with specific cell types, brain regions, and physiological or pathological states 193. For example, Hagihara demonstrated that stress-induced neuronal excitation and social defeat promote histone lactylation (especially H1Kla) in brain cells, enhancing c-Fos expression, which leads to reduced social behavior and increased anxiety 193. Similarly, Fei *et al.* found that hypoxia amplifies p53 Kla in microglia, driving LPS-induced pro-inflammatory activation of BV2 cells via the NF-κB pathway, thereby exacerbating neurodegeneration 194. He *et al.* reported that NCOA4 K450la enhances iron autophagy and glycolysis in hippocampal neurons, accelerating ischemic brain injury 195. However, some studies offer contrasting perspectives. For instance, Wang *et al.* revealed that microglial Bach1 deficiency during brain development lowers lactate levels, reducing H4K12la enrichment at the Lrrc15 promoter. This activates the JAK/STAT3 pathway, regulates astrogliogenesis, and leads to anxiety-like behaviors, including impaired exploration and social deficit 196. Wu *et al.* showed that hippocampal lactate injections upregulated PSD95, SYP, and GAP43 expression in hippocampal tissues and HT22 cells, enhancing spatial memory in mice through protein Kla 197. Additionally, Han *et al.* demonstrated that exercise-induced histone H3 lactylation in microglia transforms pro-inflammatory microglia into anti-inflammatory/repair phenotypes, mitigating neuroinflammation and improving cognitive function—similar to the “lactate clock” in macrophages 198. This indicates that exercise-induced lactate and lactylation facilitate microglial phenotype transformation, playing a key role in reducing neuroinflammation and enhancing cognitive performance 198. Yan *et al.* further revealed that physical exercise enhances synaptic structure and neuronal activity in the medial prefrontal cortex by mediating Kla of the synaptic protein SNAP91, increasing resilience to chronic restraint stress. Notably, SNAP91 Kla was essential for preventing anxiety-like behaviors in CRS mice. These findings underscore a previously unrecognized non-histone Kla mechanism in regulating neural function, highlighting the brain's metabolic adaptations during exercise 199. Overall, these studies illustrate the complex, multifaceted role of Kla in the nervous system, offering potential therapeutic targets for neurological disorders.

Alzheimer's disease (AD), the most common neurodegenerative disorder, is characterized by extracellular amyloid-β (Aβ) deposits, intracellular tau hyperphosphorylation leading to neurofibrillary tangles, and microglia-mediated neuroinflammation 200. Pan *et al.* identified elevated histone Kla levels in both clinical brain samples and mouse models of AD, with H4K12la being the most prominent, indicating its potential as a therapeutic target 200. This lactate-dependent histone modification was found at glycolytic gene promoters, activating transcription and worsening microglial dysfunction through a “glycolysis-H4K12la-PKM2” positive feedback loop 200. Deleting PKM2 in microglia reduced Aβ levels in AD mouse models and improved learning and memory by lowering glycolytic rates and suppressing H4K12la expression, thus restoring microglial function. This suggests that disrupting this feedback loop could be a viable therapeutic approach 200. Wei *et al.* observed elevated lactate levels in microglia and hippocampal tissues in aged mice and AD models (FAD4T and APP/PS1), which increased global histone Kla levels, especially H3K18la. Enhanced H3K18la activated the NFκB signaling pathway by binding to Rela and NFκB1 promoters, leading to upregulation of SASP components such as IL-6 and IL-8, further exacerbating brain aging and AD 201. Wang *et al.* reported reduced levels of isocitrate dehydrogenase 3β (IDH3β), a key tricarboxylic acid cycle enzyme, in AD patient brains and transgenic mice. Knockdown of IDH3β caused oxidative phosphorylation uncoupling, reduced energy metabolism, and lactate accumulation. Overexpressing IDH3β raised lactate levels, promoting histone Kla and increasing paired box gene 6 (PAX6) expression 202. However, PAX6, a repressor of IDH3β, further suppressed its expression, causing tau hyperphosphorylation, synaptic damage, and cognitive deficits. Breaking this cycle by upregulating IDH3β or downregulating PAX6 could potentially mitigate neurodegeneration and cognitive decline in AD.

Acute ischemic stroke (AIS) occurs when cerebral vessels are blocked, resulting in ischemia and brain tissue necrosis, which can lead to severe and permanent damage to the central nervous system 203. Zhou *et al.* showed that LRP1 helps transfer healthy mitochondria from astrocytes to neurons by reducing lactate production in astrocytes and lowering the Kla of ARF1 203. Xiong *et al.* found that increased lactate from astrocytes worsens ischemic brain injury by promoting the formation of protein Kla. Inhibiting lactate production or blocking its transfer to neurons significantly reduces protein Kla levels in ischemic brains. Likewise, lowering protein Kla levels—either by using the antagonist a-485 to inhibit p300 and block protein Kla formation or by knocking out LDHA—can greatly reduce neuronal death and astrocyte activation in cerebral ischemia, extend the reperfusion window, and improve functional recovery from ischemic stroke 204. However, it is important to note that while increased lactate during the ischemic phase may promote protein Kla formation, lactate treatment during the reperfusion phase does not affect brain protein Kla levels and has a neuroprotective effect 204. Yao *et al.* reported that after cerebral ischemia-reperfusion injury, Kla levels of key Ca2+ signaling proteins, SLC25A4 and SLC25A5, increase in rat brain endothelial cells, while Kla levels of VDAC1 decrease, suggesting that Kla may mediate neuronal death through Ca2+ signaling pathways 205. Moreover, Song *et al.* found that the traditional Chinese medicine Buyang Huanwu Decoction reduces pan-Kla and H3K18la protein levels and Apaf-1 transcriptional activity, decreasing glycolysis and apoptosis in rat brain microvascular endothelial cells, thus slowing AIS progression 206. In conclusion, recent studies are increasingly focusing on the relationship between Kla and neurological diseases, seeking ways to improve outcomes for patients with central nervous system disorders through Kla modulation.

### Skeletal system diseases

Lactylation plays a vital role in skeletal and muscular diseases. For example, Xia *et al.* observed increased H3K18la levels in osteoarthritis (OA) and identified LDHA-mediated H3K18la as regulating TPI1 gene transcription, contributing to OA progression 207. Wu *et al.* reported lower lactate levels in osteoporosis patients, noting that elevated lactate levels from exercise can induce histone Kla in mesenchymal stem cells, alleviating osteoporosis 208. Zhang *et al.* discovered decreased glutamine levels alongside increased lactate accumulation and Kla in severely degenerated nucleus pulposus tissue; glutamine supplementation was found to prevent disc degeneration by inhibiting glycolysis and reducing AMPKα Kla 209. Lin *et al.* identified lactylation sites linked to cholesterol metabolism and fascia matrix synthesis in tendon samples, concluding that increased Kla levels may signal impaired cholesterol metabolism and tendon matrix degeneration 210. Overall, modulating lactate levels and Kla presents promising therapeutic potential for skeletal diseases, warranting further investigation to identify effective treatment targets.

### Digestive system diseases

Lactylation plays a critical role in digestive diseases such as ulcerative colitis (UC) and non-alcoholic fatty liver disease (NAFLD). Research indicates that inhibiting H3K18la can mitigate UC by reducing macrophage pyroptosis, restoring intestinal immunity, and enhancing the mucosal barrier 211. The traditional Chinese medicine Ge Gen Qin Lian Tang modulates histone Kla, preventing M1 macrophage polarization and slowing UC progression 212. These findings suggest that regulating lactylation could be a safe and effective therapeutic approach for UC. In NAFLD, mitochondrial pyruvate carrier 1 (MPC1) expression is associated with increased liver lipid deposition. MPC1 regulates lactate levels by influencing pyruvate metabolism, impacting the Kla of various proteins, such as fatty acid synthase, particularly at the K673 site, thereby exacerbating hepatic lipid metabolism in NAFLD 213. Despite increased liver lactate levels following MPC1 gene knockout, liver inflammation does not occur 213. Thus, Kla modulation presents promising therapeutic strategies for these conditions. However, broader clinical studies are necessary to confirm the efficacy and safety of Kla regulation.

### Endocrine system diseases

Lactylation is closely linked to endocrine disorders. Diabetic nephropathy (DN), the most common complication of diabetes, is a leading cause of death among diabetic patients 214. Studies highlight lactate accumulation as a biomarker for DN progression 214. ACSF2 K182la disrupts mitochondrial function, leading to tubular damage in diabetes 215. Additionally, aging also shifts renal tubular epithelial cells from oxidative phosphorylation to glycolysis, increasing renal lactate levels 216. Elevated lactate enhances H3K14la in diabetic kidney disease (DKD), upregulating KLF5 expression, which suppresses CDH1 transcription and accelerates EMT 216. Reducing lactate and Kla levels could significantly mitigate tubular fibrosis in DKD patients.

Chronic kidney disease (CKD) now affects over 10% of the global population, posing a serious public health concern 217. Research shows that the glycolytic enzyme PFKFB3 drives abnormal histone Kla levels in renal tubular epithelial cells. Targeted PFKFB3 knockout reduces H4K12la, suppresses NF-κB pathway activation, and alleviates renal inflammation and fibrosis, providing promising strategies for CKD prevention and treatment 218.

### Others

Lactylation is closely associated with retinal diseases. For instance, Huang *et al.* found that YY1 Kla regulates the transcription of inflammatory genes such as STAT3, CCL5, IRF1, IDO1, and SEMA4D, promoting microglial activation, migration, and proliferation, which contributes to microglial dysfunction in autoimmune uveitis 17. This study identifies p300 as the “writer” of YY1 Kla, and suggesting that targeting the lactate/p300/YY1 Kla/inflammatory gene axis could have therapeutic potential 17. Wang *et al.* further revealed that under hypoxic conditions, non-histone YY1 undergoes K183la in microglia, upregulating FGF2 expression and promoting retinal neovascularization. This highlights the lactate/p300/ YY1 Kla/FGF2 axis as a potential therapeutic target for proliferative retinal diseases 219.

In pregnancy-related diseases, Kla also plays a significant role. For example, Li *et al.* found that reduced uteroplacental blood flow in preeclampsia leads to placental hypoxia, which induces excessive lactate production in trophoblasts, triggering histone lactylation. This process regulates the expression of genes associated with placental fibrosis in preeclampsia, such as FN1 and SERPINE1, providing new insights into placental dysfunction mechanisms 220. Similarly, Huang *et al.* observed significantly elevated lactate and histone lactylation levels in patients with gestational diabetes mellitus (GDM). Histone Kla modifications in the GDM group prominently affected promoter regions, with CACNA2D1 identified as a key gene. This gene influences GDM progression by promoting cell viability and proliferation 221.

Lactylation is also important in skin injury and disease progression. For instance, Liu *et al.* reported elevated histone Kla levels in hypertrophic scar tissues and fibroblasts. Histone Kla enhances the transcriptional activity of SLUG while inhibiting PTEN, suppressing autophagy, and promoting collagen deposition and cell viability, thus regulating hypertrophic scarring 222. Zhao *et al.* found reduced levels of ADIPOQ and H3K18la in psoriasis skin tissues. Lactylation promotes the binding of ADIPOQ to the H3K18la promoter region, and the downregulation of H3K18la inhibits psoriasis progression by suppressing ADIPOQ 223.

## Lactylation detection technology and its applications in diagnosis

Recent advancements in technologies like affinity enrichment, multidimensional separation, and biospectrometry have opened new avenues for Kla proteomics development. High-performance liquid chromatography has become a standardized separation technique in proteomics. The detection of cyclic immonium ions from lactyl lysine via MS is a reliable marker for identifying lactylated peptides, as verified through affinity enrichment lactyl proteome analysis and evaluation against nonlactylated spectral libraries 11. This approach encompasses affinity enrichment, targeted lactylation proteomics, and large-scale bioinformatics assessments of nonlactylated protein databases. Using these methods, researchers like Zhang *et al.* and Wan *et al.* identified novel lactylations, revealing widespread lactylation in the human proteome 3, 11. Besides MS, immunodetection methods are frequently employed. For instance, a ynoic functionalized chemical reporter molecule called YnLac has been developed for detecting protein lactylation in mammalian cells, specifically targeting nonhistone proteins 42. However, the specificity and sensitivity of such fluorescent probes for large-scale detection require further refinement.

Artificial intelligence-based analysis is increasingly employed for lactylation prediction and study. Tools like FSL-Kla (http://kla.zbiolab.cn/) predict Kla sites computationally, aiding in systematic analysis and predicting various PTM sites 224. Additionally, Lai *et al.* developed an automated machine learning model, Auto-Kla, which demonstrates stable and accurate performance in predicting PTM sites. This model is accessible via http://tubic.org/Kla and https://github.com/Tubic/Auto-Kla 225. In summary, with ongoing technological improvements, more sensitive and high-throughput methods are expected to enhance lactylation detection and analysis accuracy.

Additionally, research also highlights lactate's multiple metabolic pathways, making it ideal for clinical metabolic imaging 226. Hyperpolarized [1-13C] pyruvate magnetic resonance spectroscopy can noninvasively detect pyruvate metabolism in real-time, with the lactate signal or pyruvate/lactate ratio correlating to lactate production 226. This method enables clinical mapping of lactate production and enzymatic transformation on subminute timescales, particularly under the Warburg effect 226. Currently, hyperpolarized lactate signals, combined with metabolic indices like 18F-fluorodeoxyglucose-positron emission tomography, are utilized in clinical diagnoses and prognoses, providing insights into tumor burden, staging, and invasiveness 227. Since 2013, hyperpolarized [1-13C] pyruvate probes have been FDA-approved for prostate cancer treatment 228. Beyond oncology, these probes are used in preclinical studies of metabolically active noncancerous tissues such as the heart, liver, and central nervous system 226. Preclinical evidence suggests their potential for organ transplant preservation research when coupled with magnetic resonance and normothermic perfusion 226.

In summary, lactylation offers promising therapeutic opportunities. Before disease onset, interventions to modulate lactylation through diet, exercise, or lifestyle changes could enable early prevention 1. During disease progression, particularly in tumors, metabolic disorders, and inflammatory diseases, altered lactylation levels may serve as biomarkers for early diagnosis and monitoring. Personalized treatment plans, informed by lactylation variability, could improve therapeutic outcomes and minimize side effects 226. Finally, identifying key lactylated proteins and enzymes may lead to targeted interventions through small molecules or biologics, paving the way for innovative disease treatments.

## Challenges and Perspectives

Since its discovery in 2019, Kla has attracted significant attention for its potential role in regulating various physiological and pathological processes. The identification of Kla has expanded our understanding of glucose metabolism, providing new perspectives for exploring biological functions and developing therapeutic strategies 29. Despite existing research, the study of Kla remains in its early stages, with several key areas still unexplored.

First, while Kla primarily originates from the intracellular metabolite lactate, it is unclear whether it is an inevitable consequence of lactate accumulation. The full range of lactylation substrates also remains challenging to identify. Second, although Kla refers to the covalent linkage of a lactyl group to lysine residues, it is still unknown whether Kla can occur on other amino acids, such as glycine, cysteine, or serine. Third, KL-La is a reversible process, influenced by specific enzymes. However, the key enzymes responsible for these processes are still debated, particularly due to the lack of data on substrate specificity and kinetic parameters. Fourth, the interactions between Kla and other PTMs, including competition, coordination, or selection mechanisms, remain unclear. Finally, while Kla is recognized as a critical regulatory factor in tumor development and immunotherapy, its epigenetic regulatory networks and molecular mechanisms in physiological and pathological processes require further investigation.

Research has also highlighted the challenge of maintaining lactylation levels within physiologically and pathologically relevant ranges 29. An inducible orthogonal translation system using pyrrolysyl-tRNA synthetase offers a promising method for selectively increasing lactylation levels in a dose-dependent manner, with fewer off-target effects. However, this approach requires complex genetic manipulations and lacks precise control over lactylation levels, often resulting in artificial increases that may not reflect true pathophysiological conditions. Current assays used to study lactylation frequently involve experimental conditions that may not accurately represent *in vivo* environments. For instance, the concentrations of lactoyl-CoA used in some studies are much higher than those found in cells, potentially distorting results and overestimating lactylation's role in cellular processes. Lactate supplementation or LDHA inhibition affects other cellular processes, such as redox balance and immune regulation. Inhibiting upstream regulators of glycolysis could have even broader side effects, raising questions about the specificity and efficiency of lactylation regulation. Additionally, the lack of targeted tools for manipulating lactylation, such as enzymes that selectively affect lactylation without impacting other biological processes, limits the ability to study its functions independently. Thus, developing methods to control lactylation levels under pathophysiological conditions is crucial for gaining clearer insights into its role in cancer.

Currently, targeting lactate and lactylation metabolism is a promising therapeutic strategy (Table 4). However, clinical approaches targeting Kla, including potential combination therapies, still require validation through clinical trials. Future research should focus on elucidating the mechanisms of lactylation, systematically analyzing its regulatory networks, developing precise detection tools, and evaluating its safety and efficacy as a therapeutic target.

## Conclusion

Lactylation, an important form of PTMs, has emerged in recent years as a key player in health and disease. Although research on its physiological and pathological roles is still in its early stages, evidence highlights its significance in pathology. The regulation of Kla is expected to become a pivotal focus in developing new therapeutic strategies for major diseases, such as cancer. Identifying specific lactylation targets and integrating them with high-throughput detection technologies could facilitate early diagnosis and disease classification. Targeting lactylation-related enzymes, such as lactoyltransferases and delactylases, or modulating lactate metabolism, could enable more precise drug interventions. Clinically, therapies targeting lactylation could offer new options for cancer treatment, improving both efficacy and quality of life. Drug delivery systems with lactose glycosylation-specific structures could boost drug accumulation at tumor sites, increasing treatment efficiency. Combining immunotherapy with metabolic regulation strategies may further establish lactylation as a key component of combination therapies. In summary, lactylation research not only provides new insights into disease mechanisms but also holds significant potential for innovative therapeutic development. As the field evolves, lactylation could transition from research to clinical applications, paving the way for breakthroughs in health and disease management.

## Figures and Tables

**Table 1:** Histone lactylation sites identified in diverse species.

Histone	Kla Site	Species	Function	Ref.
H2A	11, 13, 115	homo sapiens	unknown	PMID: 31645732
	4, 9, 11, 115	mice	unknown	PMID: 31645732, PMID: 35685793
	5, 21, 116	Trypanosoma brucei	unknown	PMID: 34722503
	5, 137, 142	Tachyzoite	unknown	PMID: 35610722
H2A. Z	32, 36, 44, 165	Trypanosoma brucei	unknown	PMID: 34722503
	5, 9, 17, 23, 142, 150	Tachyzoite	unknown	PMID: 36216028
H2A1	73	Tachyzoite	unknown	PMID: 35610722
H2AX	127	Tachyzoite	unknown	PMID: 35610722
H2B	5, 6, 11, 15, 16, 20, 23, 43, 85, 108, 116, 120	homo sapiens	unknown	PMID: 31645732, PMID: 37919786
	5, 11, 15, 16, 20, 85, 108	mice	unknown	PMID: 31645732
	5, 97	Trypanosoma brucei	unknown	PMID: 34722503
	37, 47, 70, 77, 99	Tachyzoite	unknown	PMID: 35610722
	15, 48, 122	Botrytis cinerea	unknown	PMID: 33193272
	41, 60, 66, 114, 136, 144	Paddy	unknown	PMID: 34264677
H2Bv	8, 20, 28	Trypanosoma brucei	unknown	PMID: 34722503
H2BZ	3, 8, 18, 104	Tachyzoite	unknown	PMID: 36216028
H2Bb	46, 98	Tachyzoite	unknown	PMID: 36216028
H3	9, 14, 18, 23, 24, 33, 56, 62, 79, 123	homo sapiens	H3K9: promote muscle generated; temozolomide resistance in glioma; regulates CD8 T cell effector functions, including anti-tumor immunity; H3K9, H3K14, H3K56: Promote liver cancer cell proliferation and metabolic reprogramming; H3K18: Cell differentiation and metabolic reprogramming; Promote tumor progression; Promote bevacizumab/ cisplatin resistance; Potential biomarkers for diagnosing; Aggravate brain aging and Alzheimer's disease; Promote pulmonary fibrosis; Aggravating cerebral ischemia-reperfusion injury; Regulating effect of CD8 T cell functions; Cause inflammation and aggravate renal function damage; Activating transcription of TTK and BUB1B; Improves atherosclerosis;	PMID: 31645732, PMID: 35605812, PMID: 36192798, PMID: 37615625, PMID: 38670996, PMID: 38901791, PMID: 38854142, PMID: 38769664, PMID: 38767134, PMID: 38733581, PMID: 38711083, PMID: 38477507, PMID: 38443347, PMID: 37919243, PMID: 38295753
	18	Sheep	unknown	PMID: 34861240
	14, 18, 23, 27, 56	mice	H3K18: Promote angiogenesis after myocardial infarction, improves cardiac function after myocardial infarction; Aggravate brain aging and Alzheimer's disease; H3K23: Participate in pre-implantation development	PMID: 31645732, PMID: 34930415, PMID: 38232735, PMID: 37697347
	24, 33, 62	Trypanosoma brucei	unknown	PMID: 34722503
	14, 23, 27, 56, 122	Tachyzoite	unknown	PMID: 35610722, PMID: 36216028
	123	Botrytis cinerea	unknown	PMID: 33193272
	9, 14, 18, 56	Paddy	unknown	PMID: 34264677
H4	5, 8, 12, 16, 31, 77, 80, 91	homo sapiens	H4K5, H4K8: Regulation of telomerase activity; H4K5, H4K8, H4K12: Promote tumor progression and drug resistance; H4K12: Promote inflammation; A novel biomarker for Triple-negative breast cancer; Potential targets for the treatment of Alzheimer's disease; Promotes anaplastic thyroid carcinoma; Accelerate DNA replication and the cell cycle;	PMID: 31645732, PMID: 37919786, PMID: 34150616, PMID: 35959377, PMID: 35303422, PMID: 37777506, PMID: 38844063, PMID: 38779451, PMID: 37184950, PMID: 37587486
	8, 12, 31, 91	mice	glycolysis-related genes; Promotes inflammatory infiltration in the microenvironment;	PMID: 31645732, PMID: 35303422 PMID: 36430853, PMID: 38486199
	78	Trypanosoma brucei	unknown	PMID: 34722503
	12, 31	Tachyzoite	unknown	PMID: 35610722, PMID: 36216028

**Table 2:** Nonhistone lactylation sites identified in diverse species.

Species	Nonhistone	Kla Site	Function	Ref.
Homo sapiens	β-catenin	Unknown	Hypoxia-Wnt signaling and promote the protein stability;	PMID: 36464122
	PDHA1	K336	Inflow of acetyl-CoA that restricts pyruvate oxidation to inactivate enzymes and inhibit OXPHOS;	PMID: 38163844
	PTBP1	K436	The proteasomal degradation of PTBP1 is inhibited by weakening its interaction with TRIM21;	PMID: 39570804
	PD-L1	unknown	It is associated with the mechanisms of immune evasion in cytotoxic T cell-mediated tumor immunity;	PMID: 39577415
	NLRP3	K245	LDHA promotes myocardial I/R injury by enhancing the lactylation of NLRP3, thereby inducing cardiomyocyte pyroptosis.	PMID: 39548367
	Hdac1	unknown	Promote the removal of H3K27ac and the silencing of the 2-cell (2C) gene;	PMID: 39533309
	Tufm	K286	Tufm-mediated mitophagy is inhibited, while mitochondrial-induced neuronal apoptosis is increased;	PMID: 39496783
	YTHDF1	unknown	The virulence factor NSs of SFTSV can promote the liquid-liquid phase separation and degradation of YTHDF1, thereby enhancing the replication of SFTSV	PMID: 39496835
	P4HB	K311	Stabilize the kynurenine metabolism mediated by GOT2;	PMID: 39494721
	TFEB	K91	Emulsification of K91 prevents the interaction between TFEB and the E3 ubiquitin ligase WWP2, thereby inhibiting TFEB ubiquitination and proteasomal degradation;	PMID: 39196068
	CREB	K136	hCG-induced luteinization of GCs under hypoxic conditions.	PMID: 39322166
	ACLY	K918/995	It altered its affinity for metabolic substrates and consequently modulated its metabolic activity;	PMID: 39251781
	TWIST1	K150	Promote Twist1 phosphorylation and nuclear translocation, thereby regulating the transcription of TGFB1 to induce a fibrotic phenotype.	PMID: 39474980
	c-Myc	unknown	HSPA12A increases glycolysis-derived lactate production in a HIF1α-dependent manner, thereby promoting the lactylation of c-Myc;	PMID: 39277835
	IGF2BP3	unknown	Reprogram serine metabolism and enhance the antioxidant defense system;	PMID: 39450426
	ALKBH5	unknown	Lactylated ALKBH5 binds to interferon-β (IFN-β) messenger RNA (mRNA), leading to its m6A demethylation and promoting the biosynthesis of IFN-β mRNA;	PMID: 39413129
	DHX9	K146	Alleviated the inhibitory effect of DHX9 on OSCC.	PMID: 39407253
	α-tubulin	K40	Enhanced microtubule dynamics, promoting the growth and branching of neurites;	PMID: 39333081
	SNAP91	unknown	By enhancing synaptic structural formation and neuronal activity in the medial prefrontal cortex (mPFC), it strengthened resilience to chronic restraint stress (CRS);	PMID: 39163863
	CPT2	457/8	Inflow of acetyl-CoA that limits fatty acid oxidation to inactivate enzymes and inhibit OXPHOS;	PMID: 38163844
	YY1	K183	Upregulate FGF2 transcription and promotes angiogenesis;	PMID: 37085894
	AK2	K28	Inhibition of enzyme activity;	PMID: 38750680
	MOESIN	K72	Enhance TGF- β signaling in Treg cells;	PMID: 35732125
	eEF1A2	K408	Enhance translation elongation rate and protein synthesis, thereby promoting tumorigenesis;	PMID: 38359291
	DCBLD1	K172	promote its expression, suppresses the autophagic degradation of G6PD in itself, and activates the pentose phosphate pathway (PPP) to promote cervical cancer progression;	PMID: 38291438
	MRE11	K673	Promote its binding to DNA and promote DNA end resection and homologous recombination;	PMID: 38128537
	AMPKα	unknown	Unknown;	PMID: 38486093
	Snail1	unknown	RHOF overexpression promotes the lactylation and nuclear translocation of Snail1. Silencing Snail1 reverses the promoting effects of RHOF and lactate on cell migration, invasion, and EMT;	PMID: 36735787 PMID: 9462429
	AARS1	unknown	Form a positive feedback loop with YAP-TEAD to promote gastric cancer (GC) cell proliferation; catalyze the formation of lactate-AMP and then transfer lactate to lytic receptor residues;	PMID: 38512451 PMID: 38653238
	NCL	K447	Bind to the primary transcript of the MAP kinase-activated death domain protein (MADD) and ensures efficient translation of MADD by circumventing alternative splicing that generates premature stop codons;	PMID: 38679071
	ACSF2	K182	Result in mitochondrial dysfunction;	PMID: 38676722
	Esrrb	K228/232	In the absence of LIF and XEN differentiation, lactoylation of Esrrb enhances its activity to promote ESC self-renewal by increasing its binding at the target genes;	PMID: 38473939
	ALDOA	K147	Inhibition of protein activity;	PMID: 35761067
	SHMT2	unknown	Unknown;	PMID: 38175377
	G6PD	K457	Reexpression in the HPV16-positive SiHa cell line inhibited cell proliferation;	PMID: 38457903
	NEDD4	K33	Inhibition the interaction with Caspase-11;	PMID: 38385085
	NMNAT1	K128	Enhance its nuclear localization and maintained the enzymatic activity, thereby supporting the nuclear NAD rescue pathway and promoting cancer growth;	PMID: 38467179
	PFKP	K668	Inhibition of protein activity;	PMID: 38155775
	METTL16	K229	Increase protein activity;	PMID: 37863889
	P53	unknown	Development of enhanced Enz resistance and progression of PCa;	PMID: 38880227
	SLC16A1	unknown	Promoting L-lactate transport through proton junctions in the plasma membrane;	PMID: 38817665
	Ezrin	K263	Activating the NF-κB pathway and enhance the protein level of GLUT1;	PMID: 38767134
	kzf1	164	Directly regulate the expression of TH17-related genes (Runx 1, Tlr 4, IL-2, and IL-4), promoting TH17 differentiation;	PMID: 37851814
	ARF1	K73	Promote vesicle formation;	PMID: 38906140
	ENO1	K343	Affect the substrate-enzyme interactions;	PMID: 35761067
	DHRS7	K321	Unknown;	PMID: 35761067
Mice	METTL3	K281/345	Promote the capture of RNA capability;	PMID: 35320754
	tau	K677	It may prevent AD by affecting ferroautophagy and ferroptosis through the MAPK signaling pathway.	PMID: 39307193
	MDH2	unknown	Lactylation of MDH2 can induce ferroptosis by impairing mitochondrial function, leading to MIRI (myocardial ischemia-reperfusion injury;	PMID: 39467114
	CIRP	unknown	Leads to the release of CIRP;	PMID: 39465383
	NCOA4	K450	Enhanced the stability of the NCOA4 protein;	PMID: 39166386
	HMGB1	unknown	Promote translocation of nucleus to cytoplasm and release through exosomes;	PMID: 34363018
	PKM2	K62	Enhance enzyme activity and reduces nuclear distribution, and induces cell cycle arrest in S phase	PMID: 36439872 PMID: 38570082
	LCP1	unknown	Promote the protein stability;	PMID: 36574182
	FASN	K673	Inhibition of protein activity;	PMID: 36651176
	IKZF1	K164	Promote the TH 17 differentiation;	PMID: 37851814
	α-MHC	K1897	Promote its interaction with myxin;	PMID: 37443257
*E. coli*	PykF	K382	Inhibition of protein activity;	PMID: 36333310 PMID: 37159428
	BGN	Unknown	Associated with the onset of tendinopathy;	
	TPM3	Unknown		
	MYL3	Unknown		
	COMP	Unknown		
	TNNC1	Unknown		
	FGA	Unknown		
	POSTN	Unknown		
	LUM	Unknown		
	MMP3	Unknown		
	DCN	Unknown		
	APOA1	Unknown	Involved in cholesterol metabolism;	
	APOA4	Unknown		
	APOC1	Unknown		
	APOC3	Unknown		
*Toxoplasma* *planus*	MIC1	K157	Invasion and survival;	PMID: 36216028
	MIC2	K630		
	RON6	K1170		
	ROP9	K79/138/144/347		
	ROP18	K202		
	GRA12	K74		
	CDPK1	K50/59/80/93/341	Invasion, intracellular development and propagation;	
	CDPK2A	K165/729/777		
	CDPK9	K5		

**Table 3:** Lactylation modification sites, functions and Mechanism in malignant tumors.

Tumor types		Site	Mechanisms and effects	Ref.
Lung Cancer	NSCLC	IGF1R	Promotes the proliferation and metabolic reprogramming.	PMID: 38840891
		Histone Kla	Maintain mitochondrial homeostasis, Modulates cellular metabolism	PMID: 34150616
		APOC2 K70	Induces tumor metastasis, and resistance to immunotherapy	PMID: 38981044
		H3K18la	Enhances immune evasion via the POM121/MYC/PD-L1 pathway	PMID: 39137401
		SOX9	Promotes NSCLC stemness, migration, and invasion through glycolysis	PMID: 38226837
		H3	H3la-PKM2-tumorigenesis	PMID: 38149461
		H4K12la	AKR1B10 promotes H4K12la and activates CCNB1 transcription contributing to PEM resistance	PMID: 37587486
	LUAD	H3K14la, H3K18la	Promotes the proliferation and migration of LUAD endothelial cells by downregulating SLC25A29	PMID: 37775731
		IDH3γ Kla	BZW2 promotes LUAD progression through glycolysis-mediated lactate production and IDH3γ Kla	PMID: 37955350
		H4K8 and H4K16 Kla	LKB1 inhibits H4K8 and H4K16 Kla reduces telomerase activity and promotes LUAD cell senescence	PMID: 38844063
Colorectal cancer		H3K18la	METTL3-JaK1m6A-STAT3-Immunosuppression	PMID: 35320754
			Promotes RUBCNL expression, exacerbating resistance to bevacizumab treatment	PMID: 37615625
			H3K18la- GPR37 -Hippopathway- liver metastases	PMID: 37749229
			Activates the transcription of NSUN2 and NSUN2 K356la, promoting CRC progression	PMID: 38769664
			Inhibits the transcription of the RAR γ gene and conferring macrophage protumor function	PMID: 38245869
			Increases the upregulation of CXCL1 and CXCL5 expression, ultimately promoting CRC liver metastasis.	PMID: 37749229
		H4K8la	Upregulates LINC00152 and promotes cancer cells invasion and migration	PMID: 35959377
		eEF1A2 K408la	KAT8 promotes CRC development through Kla of eEF1A2	PMID: 38359291
		β-catenin	Hypoxia-induced β -catenin Kla promotes CRC proliferation through the Wnt signaling pathway	PMID: 36464122
		MRE11 K673	Promotes DNA end resection and homologous repair	PMID: 38128537
		Protein Kla	Inhibiting M2 macrophage polarization to inhibit CRC progression	PMID: 36242053
		RIG-I Kla	affects the immunosuppressive activity of Tregs and the antitumor activity of CD8+ T cells	PMID: 38890429
Liver cancer	HCC	H3K9la, H3K56la	Upregulates ESM 1 to promote the malignant phenotype, tumor growth, and metastasis of HCC cells	PMID: 39016629
		CCNE2 Kla	Promotes proliferation, migration, and invasion through lactylation	PMID: 36896611
		CENPA K124la	CENPA is cooperated with YY1 to drive CCND 1 and NRP2 expression to promote HCC progression	PMID: 37928273
		AK2 K28la	Inhibits AK2, leading to intracellular energy disorders, cell proliferation, invasion and metastasis	PMID:36593272
		PKM2 K505la	Enhanced the immunosuppressive microenvironment and HCC metastasis	PMID: 38471282
		MOESIN K72la	Regulates regulatory T cells and exerting an immunosuppressive function to promote HCC progression	PMID: 35732125
		IGF2BP3	Reprogram serine metabolism and enhance the antioxidant defense system	PMID: 39450426
		GPC3 Kla	Promotes cell growth, stemness, and glycolysis	PMID: 37131292
		c-Myc	Reduced the stability of the c-myc protein;	PMID: 37131292
		H3K9la, H3K56la	Stimstimulates cell proliferation to promote HCC progression	PMID: 35605812
		H3K18la	PYCR1 regulates the transcriptional activity of IRS1 by affecting the lactylation of H3K18 in the IRS1 promoter region.	PMID: 39422696
		H3K56la, ALDOA K230/322	Closely related to the stemness of LCSCs	PMID: 39099416
	iCCA	NCL K477la	Up-regulation of MADD enhances the pathogenesis of intrahepatic cholangiocarcinoma through the MAPK pathway	PMID: 38679071
Breast Cancer		H3K18la	Upregulates c-Myc and promote breast cancer progression	PMID: 37572497
		p53 K120la, p53 K139la	Decreases transcriptional activity, promoting tumorigenesis	PMID: 38512451
		H4K12la	H4K12la is significantly upregulated in triple-negative breast cancer (TNBC) and is a novel biomarker.	PMID: 38779451
Gastric Cancer		H3K9la H3K18la, H3K56la	Knockdown of GLUT3 significantly reduces the levels of LDHA, L-lactyl, H3K9, H3K18, and H3K56.	PMID: 38041125
		H3K18la	Upregulates VCAM 1 transcription, activates AKT-mTOR signaling pathway and promotes tumor cell proliferation, EMT transformation, and tumor metastasis; Upregulate the expression of METTL14, which activates the WDR74/β-catenin axis by mediating the m6A modification of ATF5 mRNA, thereby promoting stemness in gastric cancer.	PMID: 39497511 PMID: 38512451
		CBX3 K10la	Promotes the tumor proliferation and growth	PMID: 39018247
		METTL16 K229la	Copper stress promotes METTL16 lactlation, and subsequently triggered copper death	PMID: 37863889
Pancreatic cancer	PAAD	SLC16A1 Kla	Promotes tumor progression	PMID: 38817665
		NMNAT1 K128la	Supporting the nuclear NAD+ salvage pathway and promoting PAAD tumor growth	PMID: 38467179
	PDAC	H3K18la	Activates TTK and BUB1B transcription, and increased glycolysis aggravates PDAC dysfunction	PMID: 38711083
		Snail1	RHOF overexpression promotes the lactylation and nuclear translocation of Snail1. Silencing Snail1 reverses the promoting effects of RHOF and lactate on cell migration, invasion, and EMT;	PMID: 39462429
		CENPA	CENPA may become a promising therapeutic target for PDAC;	PMID: 39456925
		NUSAP1K34	Forms a NUSAP1-LDHA-glycolytic-lactate feed-forward loop, thus accelerating the PDAC transfer	PMID: 37354982
Esophageal cancer		SHMT2 Kla	Increasing MTHFD1L expression, accelerates the EC malignant progression	PMID: 38175377
		Axin Kla	Promotes the ubiquitination of Axin 1, exerts its anti-glycolytic function	PMID: 38972426
		H3K9la	Enhances the transcription of LAMC 2 to promote the proliferation and invasion of ESCC	PMID: 38989468
Prostate cancer	PCa	HIF1α Kla	Enhances the transcription of KIAA1199, and promote angiogenesis.	PMID: 36209908
		p53 Kla	p53 Kla via the NF-κB/STAT3/SLC4A4 axis, leading to Enz resistance and PCa progression	PMID: 38880227
		CNPY3 K215la and K224la	Inhibits lysosome-dependent CatB/caspase 1/GSDMD pyroptosis signaling pathway, promotes PCa progression	PMID: 38511243
	NEPC	Pan-Kla, H3K18la	Zeb1 drives histone lactylation modifications to regulates the development of neuroendocrine prostate cancer.	PMID: 38654072
		Pan Kla and H3K18la	Numb/Parkin pathway Defects, accelerate metabolic reprogramming, accelerating NEPC progression	PMID: 36724072
Cervical Cancer		G6PD K45la	HPV16 E6 inhibits the Kla of the G6PD dimer by activating PPP and promotes cell proliferation	PMID: 38457903
		H3K18la	Promote the reprogramming of tumor-associated macrophages;	PMID: 39504115
		PPP1R14B K140la	Enhanced the proliferation and migration capabilities of cervical cancer	PMID: 39025375
		DCBLD1 K172la	Upregulates DCBLD1 expression, and then upregulates G6PD to stimulate PPP	PMID: 38291438
Bladder cancer		H3K18 la	circXRN2 inhibited H3K18la-driven tumor progression in BCa by stabilizing LATS1 and activating the Hippo pathway	PMID: 37684641
			H3K18la-driven YBX1 and YY1 promote BCa cisplatin resistance	PMID: 38295753
Renal-cell carcinoma		H3K14la, H3K18la and H3K56la	Overactive Warburg effects, and modulates sensitivity to HIF 2 α blockade	PMID: 38233415
		Pan Kla and H3K18la	High levels of Kla indicated poor prognosis.	PMID: 35637958
		H3K18la	Activates PDGFRβ gene transcription; PDGFRβ signaling further stimulates H3K18la to form an oncogenic positive feedback loop"	PMID: 35637958
Glioblastoma		VEGFR2 and VE-cadherin Kla	LDHA binds and promotes the lactfication of VEGFR2 and VE-cadherin and promoting the development of angiogenic mimicry in glioblastoma	PMID: 37853052
		Pan Kla and H3K18la	Driving NF- κ B-associated LINC01127 expression via the MAP4K4 / JNK / NF- κ B axis promotes self-renewal in GBM cells	PMID: 38084701
		PTBP1 K436la	Induced abnormal epigenetic modifications further stimulate glycolysis, leading to a vicious cycle that exacerbates tumorigenesis	PMID: 39570804
		c-Myc	Dexmedetomidine inhibits the lactylation of c-Myc and suppresses the stability of c-Myc;	PMID: 39193894
		H3K18la	Hypoxia can regulate TNFSF9 expression through the MCT-1/H3K18La signaling pathway, inducing M2 macrophage polarization and promoting the malignant progression of gliomas	PMID: 39010835
		XRCC1 K247la	Induces treatment resistance in glioblastoma	PMID: 39111285
		H3K9la	Retention of the MLH 1 intron 7 mediated by LUC7L2 confers resistance to TMZ in the GBM	PMID: 38477507
Uveal melanoma		H3K18la	Induces YTHDF2 overexpression accelerates PER 1 and TP53 mRNA degradation and promoting the onset of UM	PMID: 33726814
		Pan Kla, H3K18la	Histone lactlation enhances ALKBH3 expression through demethylation of m1A of SP100A	PMID: 38118002
Other	Lymphadenoma	Kla	Elevated lactate levels in lymphoma patients influence the lactylation status, which in turn affects the prognosis, tumor immune function, and drug resistance of diffuse large B-cell lymphoma.	PMID: 39146596
	oral squamous cell carcinoma	DHX9 K146la	Alleviated the inhibitory effect of DHX9 on OSCC;	PMID: 39407253
	Endometrial cancer	H3K9la, H3K18la, H3K28la,	Promotes USP39 expression, and targeting the PI3K/AKT/HIF-1α signaling pathway promotes the malignant progression of EC	PMID: 38459014
		H3K18la	Both aerobic glycolysis and histone lactfication were increased	PMID: 38744310
	Anaplastic Carcinoma	H4K12la	Dysregulation of cell-cycle-related gene expression	PMID: 37184950
	Ovarian cancer	H3K18la	Lactate activates CCL18 expression through H3K18la in macrophages, promoting the OC occurrence	PMID: 39010846

NSCLC: Non-small cell lung cancer; LUAD: Lung adenocarcinoma; HCC: Hepatocellular carcinoma; iCCA: Intrahepatic cholangiocarcinoma; PAAD: Pancreatic cancer; PDAC: Pancreatic ductal adenocarcinoma; PCa: Prostate cancer; NEPC: Neuroendocrine prostate cancer.

**Table 4:** Lactate-Lactylation in Malignancy and treatment.

Therapeutic targets and methods	Effects	Cancer	Object	Representative drugs or antibody	Ref/Trial No.
MCT1	Lactate uptake	Raji B cell lymphoma	Human	AZD3965	NCT01791595
		Triple negative breast cancer	Human	BAY8002	PMID: 33333023
		Neuroblastoma, lymphoma, breast cancer	Human	SR13800	PMID: 32123312
		Breast cancer, colorectal cancer	Cell lines, mouse	AR-C155858	PMID: 24672058
		Pancreatic cancer	Cell lines, human tissue, mouse	7ACC2	PMID: 32138176
	Regulation of Treg cells	HCC	Cell lines, human tissue, mouse	anti-PD 1	PMID: 35965549
PD-1	Regulates the cytotoxicity of CD8 + T cells	NSCLC	Cell lines, mouse	anti-PD 1	PMID: 39137401
PD-1 & MCT1/4	Through MCT1, Treg cells actively absorbed LA, which enhanced the expression of PD-1 and dampened the expression of PD-1 by effector T cells.	MYC-amplified tumors and liver tumors	Cell lines, mouse, human and human tissues	pembrolizumab or nivolumab	PMID: 35090594
	Regulates the expression of Mct4/Slc16A	Melanoma	Cell lines, mouse, human tissues	pembrolizumab and nivolumab	PMID: 32123312
	Inhibiting MCT4 can enhance the activity of CD8+ T cells	Hepatocellular carcinoma	Cell lines, mouse, human and human tissues	Toripalimab, MCT4 inhibition	PMID: 38522774
	Combination of MCT4 and ICB increased intratumoral pH, delayed tumor growth, and prolonged survival	Colorectal carcinoma	Cell lines, mouse; human blood	anti-PD L1 antibody, MCT4 inhibition	PMID: 37880183
	Lactate excretion	Myeloma	Cell lines, human tissue, mouse	Syrosingopine	PMID: 36807305
PD-1 & LDHA	Oxamate enhances the therapeutic effect of pembrolizumab.	Non-small cell lung cancer	Mouse	Oxamate, anti-PD-1 pembrolizumab	PMID: 33859941
PD-1 & LDHA	Deficiency of LDH-A increased infiltration of NK cells and CD8+ cytotoxic T cells, improving the efficacy of anti-PD-1 therapy.	Melanoma	Cell Lines; mouse	Deletion of LDHA; Anti-PD-1 antibody	PMID: 30934955
CAR-T therapy	HDAC inhibition induced by lactate enhanced CD8+ T cell exhaustion efficiently inhibit tumor growth.	Melanoma and colon adenocarcinoma	Cell lines, mouse, Human blood	Glucose or sodium lactate;	PMID: 36068198
CAR-T therapy	Lactate increase the immunogenicity of whole tumor cell vaccines that have been irradiated with UV light.	Lymphoma	Cell lines, mouse	Lactic acid; irradiation	PMID: 31734353
	Promotes immune activation of tumor-infiltrating CAR T cells.	Glioblastoma	Cell lines, mouse	Oxamate,LDH-A inhibitor	PMID: 34978120
LDHA	Lactate production	Prostate and lung adenocarcinomas	Cell lines, mouse, human tissue	Oxamate	PMID: 35075123
	Lactate uptake, restrain LDHA	liver cancer	Cell lines, mouse	Royal jelly acid	PMID: 37453194
PDHK	Lactate production	Metastatic breast cancer, lung cancer	Human	Dichloroacetate	NCT01029925, NCT01386632
Hexokinase	Lactate production	Prostate cancer, lung cancer, breast cancer, etc.	Human	2DG	NCT00096707, NCT00588185
		Breast cancer, liver cancer, bladder cancer	Cell line, mouse	3-BrPA	PMID: 32330495
		Breast cancer	Cell line	Tristetraprolin	PMID: 30650008
		Lung cancer, breast cancer, melanoma, etc.	Cell line	Lonidamine	PMID: 30472500
GPR81	Lactate uptake	Acute lymphoblastic leukemia, prostate cancer, etc.	Human	Curcumin	NCT05045443, NCT04731844
HDAC	Lactylation	Nasopharynx cancer	Cell lines	ITSA-1	PMID: 23946197
HAT		Breast cancer, colon cancer, lung cancer, etc.	Cell lines	Garcinol	PMID: 34785033
p300/CBP		Neovascularization, pituitary adenoma, melanoma, etc.	Cell lines	A-485	PMID: 30387898
GCN5		lung cancer	Cell lines, human tissue	CPTH6	PMID: 36621008
Mitochondria	Regulation of histone Kla and therapeutic cancer cell plasticity	Neuroendocrine prostate cancer	Cell lines, mouse, human tissue	Unmentioned	PMID: 36869843
	Induces mitochondrial apoptosis and regulates the levels of Kla and 2-hydroxyisobutyllation	Breast cancer	Cell lines, mouse	Catalpol	PMID: 36067808
Combined treatment	Reduces Kla levels	Endometrial carcinoma	Cell lines, mouse, human tissue	2-DG and sodium acetate	PMID: 39320546
	Reduces lactate production	Prostatic carcinoma	Cell lines, mouse	Copanlisib and ADT	PMID: 36862086
	Enhances the efficacy of bevacizumab in colorectal cancer	Colorectal cancer	Cell lines, mouse, human tissue	Kla and autophagy inhibitors	PMID: 37615625
Immunization therapy	Affects the immunosuppressive activity of regulatory T cells and the antitumor activity of CD8 + T cells	Colorectal cancer	Cell lines, mouse	Vitamin A acid	PMID: 27590114
	Reduces resistance to immunotherapy	Non-small cell lung cancer	Cell lines, mouse	anti-apoc2-k70-lac	PMID: 38981044

## Abbreviations

PTM: Protein post-translational modification
Kla: Lactylation
Kac: Acetylation
Glis1: Phosphoglycerate kinase 1
Arg1: Arginase1
Brg1: SBP (S-ribonuclease binding protein) family protein
P300: E1A binding protein p300
CBP: CREB binding protein
MCT1: Monocarboxylate transporter-1
ROS: Reactive oxygen species
NSCLC: Non-small cell lung cancer
2-DG: 2-Deoxy-D-glucose
TME: Tumor microenvironment
CRC: Colorectal cancer
LDHA: Lactate dehydrogenase A
PKM2: Pyruvate kinase M2
ALDOA: Aldolase, fructose-bisphosphate A
AARS1/AARS2: Alanyl-tRNA synthetase 1/ Alanyl-tRNA synthetase 2
ccRCC: Clear cell renal cell carcinoma
CBX3: Chromobox protein homolog 3
CAFs: Cancer-associated fibroblasts
SAKI: sepsis-associated acute kidney injury
SLE: Systemic lupus erythematosus
CVDs: Cardiovascular diseases
MI/R: Myocardial ischemia/reperfusion
AS: Atherosclerosis
EndMT: Endothelial-to-mesenchymal transition
AD: Alzheimer's disease
AIS: Acute ischemic stroke
DN: Diabetic nephropathy
GDM: Gestational diabetes mellitus
